# Characterization of noise in multistable genetic circuits reveals ways to modulate heterogeneity

**DOI:** 10.1371/journal.pone.0194779

**Published:** 2018-03-26

**Authors:** Sayuri Katharina Hortsch, Andreas Kremling

**Affiliations:** Systems Biotechnology, Faculty of Mechanical Engineering, Technical University of Munich, Garching, Germany; Universitat Pompeu Fabra, SPAIN

## Abstract

Random fluctuations in the amount of cellular components like mRNA and protein molecules are inevitable due to the stochastic and discrete nature of biochemical reactions. If large enough, this so-called “cellular noise” can lead to random transitions between the expression states of a multistable genetic circuit. That way, heterogeneity within isogenic populations is created. Our aim is to understand which dynamical features of a simple autoregulatory system determine its intrinsic noise level, and how they can be modified in order to regulate state-transitions. To that end, novel mathematical methods for the state-dependent characterization and prediction of noise in multistable systems are developed. First, we introduce the hybrid LNA, a modified version of the Linear Noise Approximation. It yields good predictions on variances of mRNA and protein fluctuations, even for reaction systems comprising low-copy-number components (e.g. mRNA) and highly nonlinear reaction rates. Furthermore, the temporal structure of fluctuations and the skewness of the protein distribution are characterized via state-dependent protein burst sizes and burst frequencies. Based on this mathematical framework, we develop graphical methods which support the intuitive design of regulatory circuits with a desired noise pattern. The methods are then used to predict how overall noise in the system can be adapted, and how state-specific noise modifications are possible that allow, e.g., the generation of unidirectional transitions. Our considerations are validated by stochastic simulations. This way, a design of genetic circuits is possible that takes population heterogeneity into account and is valuable in applications of synthetic biology and biotechnology. Moreover, natural phenomena like the bimodal development of genetic competence can be studied.

## Introduction

Cellular systems are inevitably subject to stochasticity due to random changes in environmental conditions as well as due to the molecular noise that is inherent to biochemical processes. On the one hand, the resulting fluctuations in the copy numbers of cellular components may lead to unpredictable and non-functional systems behaviour. Yet on the other, they might provide an evolutionary advantage by generating population diversity. Cells thus need elaborate mechanisms to precisely handle extrinsic and intrinsic noise. This holds especially true for gene regulation and signaling, where the impact of noise is significant as the copy numbers of involved components (genes, mRNA, regulatory proteins) are usually small.

The intrinsic noise of a typical gene expression system is mainly determined by two factors: First, by the combination of different reaction time-scales within the system, and second, by the system’s topology (e.g., the presence and type of feedback regulation).

The distribution of time-scales affects for example the emergence of *protein bursts*, which denote the pulsatile production of protein molecules: When the translation rate is large, a great number of protein molecules is translated from every (usually short-lived) mRNA molecule in a burst-like manner. This kind of bursts is common in prokaryotes [1], while mRNA bursts are prevalent in eukaryotes. The latter are generated in an analogous manner on the DNA-mRNA level [1–5].

The further propagation of noise through a regulatory network is severely influenced by its topology [6, 7]. In general, negative feedback loops tend to attenuate noise [8, 9], whereas positive feedback loops mostly promote fluctuations [10]. Interconnections of such structural motifs can lead to complex outcomes and are suitable for a more precise regulation of noise [11–14]. Again, the topological effects are complemented by the time-scales of the participating reactions. For example, fast fluctuations of a component tend to be averaged out by a comparably slow downstream reaction [15, 16].

Mechanisms for noise attenuation support controllable, almost deterministic systems behaviour [9, 16, 17]. Such mechanisms are relevant not only in natural processes, but also with regard to applications in synthetic biology and biotechnology. However, several examples exist in nature where noise is used to create diversity within a population of isogenic cells: fluctuations facilitate transient excursions or permanent transitions away from one stable state to another. Such heterogeneity can make the entirety of cells less prone to environmental stresses due to higher flexibility [18]. This is also believed to be the reason why in some bacteria like *Bacillus subtilis* and *Streptococcus mutans*, the development of competence—a genetically advantageous, but costly and risky process—occurs only in a subpopulation of cells [19, 20]. On a regulatory level, competence is initiated by random upregulation of a special autostimulatory peptide. The noise-driven switch from the inactive to the active expression state has already been studied with mathematical models [18, 21–24]. It has also been shown mathematically and experimentally that reduction of noise in the inactive state (which can be achieved by enhancing the basal transcription while reducing the translation of the peptide) impedes competence initiation [24, 25]. Now, we like to go one step further and understand how noise—despite being large in the inactive expression state—can be so small in the active expression state that competence is robustly sustained. This is not trivial since the states are connected to one another in that they originate from the same reaction network.

More generally speaking, we like to study the emergence of various noise patterns in multimodal regulatory circuits (i.e. circuits that support two or more distinct protein expression states). With the term “noise pattern”, we denote the distribution of noise levels among the expression states (e.g. large protein noise in the low expression state, small noise in the high expression state, as in competence development). Since noise levels affect transition probabilities, we can finally make qualitative predictions on population behaviour based on the circuit properties.

In this study, we focus on minimal single-gene autoregulatory systems with transcriptional feedback. Stochastic modeling is used, as random fluctuations and cell-state switches cannot be described with a deterministic model, whose behavior is uniquely defined by its initial conditions. As a first step, noise is mathematically characterized via the means and variances of mRNA and protein fluctuations. Since special emphasis is put on multimodal protein distributions (i.e. distributions with multiple peaks, each one corresponding to an expression state), it is valuable to quantify noise “locally” for every peak. One approach consists in the so-called Linear Noise Approximation (LNA) [26]. It uses a deterministic model based on rate laws, whose variables serve as approximations of the local (i.e. expression-state-specific) mean values. Then, variances are calculated after linearizing all reactions at these deterministic variables. However, when components with low copy numbers are involved, the results might be unreliable. We hence present a novel approach, called the hybrid LNA, which uses an alternative deterministic model that takes the discreteness and stochasticity of the mRNA level into account and finally leads to better approximations. As further noise measures, the cell-state-dependent protein burst size and frequency are introduced and interpreted. They help to characterize the temporal structure and skewness of the protein distribution. For all noise measures, we obtain formula that show their dependence on the circuit properties. We use them to identify properties of multimodal systems by which different noise patterns can be created, and based on these patterns, we make predictions on the behaviour of the population. Our results are verified by stochastic simulations.

## Model formulation and method development

### Formulation of a single-gene autoregulatory system

We consider mRNA and protein dynamics in a simple single-gene autoregulatory system. The reactions include transcription, translation, and mRNA and protein degradation, and can be represented as follows:

$$\begin{array}{lll} \text{reaction}\;1:\; & \emptyset & \overset{\hat{F}\left(s\right)}{\to }\;\text{mRNA}\\ \text{reaction}\;2:\; & \text{mRNA} & \overset{d_{m}}{\to }\;\emptyset \\ \text{reaction}\;3:\; & \emptyset & \overset{\hat{G}\left(m\right)}{\to }\;\text{Protein}\\ \text{reaction}\;4:\; & \text{Protein} & \overset{d_{s}}{\to }\;\emptyset \end{array}$$ (1)

**Fig 1:**
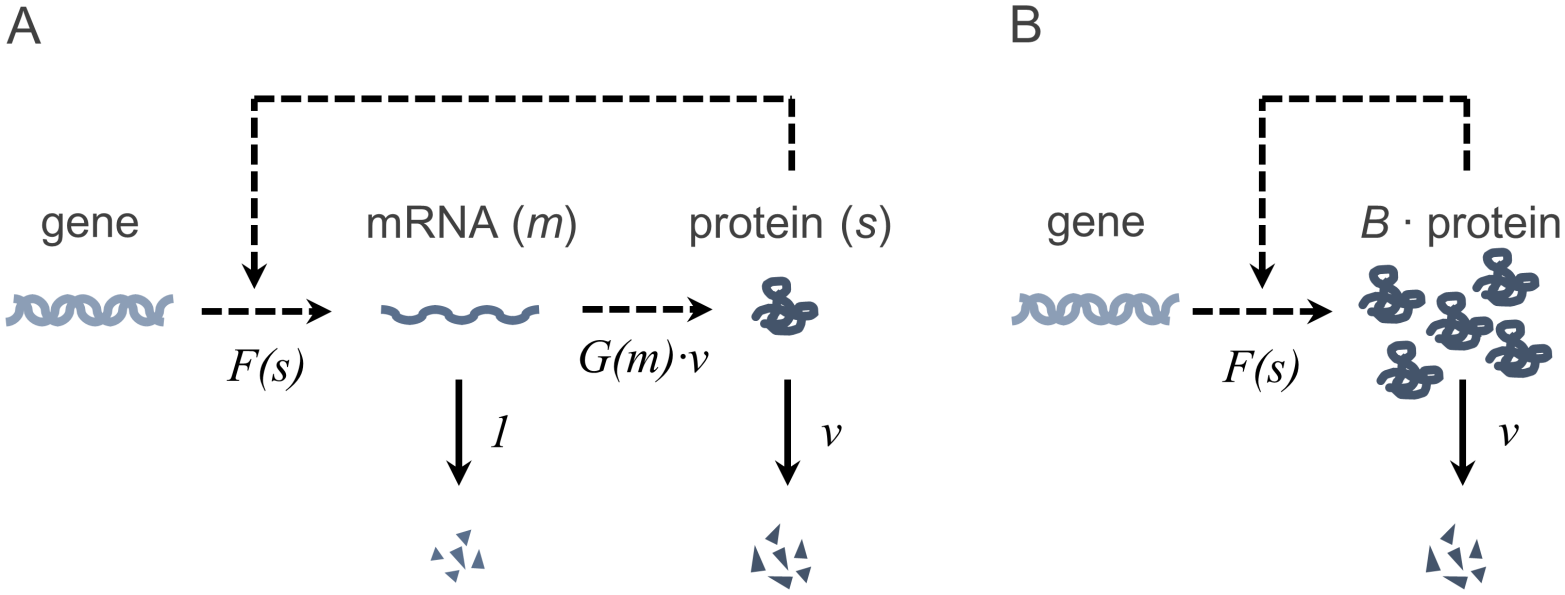
Reaction schemes of autoregulatory expression. (A): Full model including the mRNA and protein level. (B): Reduced model where the mRNA level is omitted and protein expression occurs in translational bursts of random size *B*. Solid lines indicate conversion reactions, while dashed lines describe interactions.

*m* and *s* denote the copy numbers of the specific mRNA and protein in a single cell, respectively. Autoregulation is specified by the function $\hat{F}:{\mathbb{N}}_{0}\to \mathbb{R}$, which may, depending on its monotonicity, describe a stimulatory or an inhibitory effect of the protein on its own expression. Missing feedback is modeled by constant $\hat{F}$. The strictly monotonically increasing function $\hat{G}:{\mathbb{N}}_{0}\to \mathbb{R}$, $\hat{G}(0)=0$ illustrates protein formation using mRNA as a template. The degradation rates of mRNA and protein are assumed to be linear with parameters *d*_*m*_ and *d*_*s*_. The reaction scheme is illustrated in Fig 1A.

### Stochastic model formulation

For stochastic modeling, we use the chemical master equation (see the S1 File, Sections 1.1 and 1.2, for the motivation and theoretical background). We first define the dimensionless time unit *τ* ≔ *t* ⋅ *d*_*m*_. The reference time scale is thus determined by the rate of mRNA degradation. The reaction propensities are scaled as follows: We set $F(.)≔\frac{1}{d_{m}}\cdot \hat{F}(.)$ and $G(.)≔\frac{1}{d_{s}}\cdot \hat{G}(.)$. The parameter $\nu ≔\frac{d_{s}}{d_{m}}$ relates the time scales of protein and mRNA kinetics to each other. The CME of the autoregulatory system is then given by:

$$\begin{array}{ll} \frac{dp_{m,s}\left(\tau \right)}{d\tau } & =F\left(s\right)p_{m-1,s}\left(\tau \right)-F\left(s\right)p_{m,s}\left(\tau \right)+\left(m+1\right)p_{m+1,s}\left(\tau \right)-mp_{m,s}\left(\tau \right)\\ & +\left[G\left(m\right)p_{m,s-1}\left(\tau \right)-G\left(m\right)p_{m,s}\left(\tau \right)+\left(s+1\right)p_{m,s+1}\left(\tau \right)-sp_{m,s}\left(\tau \right)\right]\cdot \nu . \end{array}$$ (2)

*p*_*m*,*s*_(*τ*) denotes the probability that at time point *τ*, *m* mRNA molecules and *s* protein molecules are present in the cell.

Usually, for general *F* and *G*, the CME cannot be explicitly solved for *p*_*m*,*s*_. It is, in many cases, even impossible to obtain explicit formula for the expectation and variance of the mRNA and protein distribution (implicit expressions are given in the S1 File, Section 2.1). Moreover, means and variances are uninformative in case of multimodal probability distributions, which are central to our study. In order to better characterize multimodality, we interpret such distributions as superpositions of unimodal ones, each having their own local mean value, variance, etc. By using the so-called linear noise approximation (LNA), closed-form estimates of these local values can be obtained.

### Deterministic model descriptions: Rate laws and hybrid model

The LNA, which will be further addressed in the subsequent section, requires the formulation of a deterministic model, whose variables serve as approximations of the local mean values of a uni- or multimodal distribution. Originally, the deterministic model is based on classical rate laws, whose variables are given in terms of concentrations and treated as continuous variables. Let *c*_*m*_ and *c*_*s*_ denote the deterministic concentrations of the regarded mRNA and protein in the cell, respectively. The scaled system of ordinary differential equations (ODEs) reads:

$$\begin{array}{ll} \frac{dc_{m}}{d\tau } & =F\left(c_{s}\right)-c_{m}\\ \frac{dc_{s}}{d\tau } & =\left(G\left(c_{m}\right)-c_{s}\right)\cdot \nu . \end{array}$$ (3)

Here, $ℱ:{\mathbb{R}}_{\geq 0}\to {\mathbb{R}}_{\geq 0}$ and $G:{\mathbb{R}}_{\geq 0}\to {\mathbb{R}}_{\geq 0}$ are the scaled transcription and translation rates in terms of concentrations. In order to obtain them, the originally discrete functions *F* and *G* are first interpolated continuously, so that they are defined on the nonnegative real line (these functions $F:{\mathbb{R}}_{\geq 0}\to {\mathbb{R}}_{\geq 0}$ and $G:{\mathbb{R}}_{\geq 0}\to {\mathbb{R}}_{\geq 0}$ will be used for further model analysis as well). After interpolation, *G* should still be strictly monotonically increasing, and if possible, the interpolation functions should be linear. Then, one sets $ℱ(c_{s})≔\frac{F(c_{s}\cdot V)}{V}\;\;\forall \;c_{s}\in {\mathbb{R}}_{\geq 0}$ and $G(c_{m})≔\frac{G(c_{m}\cdot V)}{V}\;\;\forall \;c_{m}\in {\mathbb{R}}_{\geq 0}$ [27]. *V* denotes the cell volume, which is assumed to be constant throughout the study.

In general, this deterministic formulation gives a good description of the system in case the copy numbers of all involved reaction species and the system volume are large so that fluctuations become negligible. However, these conditions do usually not hold for mRNA copy numbers in single cells. In order to circumvent this problem, we propose an alternative model which we refer to as the *hybrid deterministic model*, since it takes into account the discreteness and the stochasticity of the mRNA level although being fully deterministic. It reads:

$$\begin{array}{ll} \frac{dc_{m}}{d\tau }=F\left(c_{s}\right)-c_{m} & \\ \frac{dc_{s}}{d\tau }=\left(\bar{G}\left(c_{m}\right)-c_{s}\right)\cdot \nu , & \bar{G}\left(c_{m}\right)=\frac{1}{V}\sum_{n=0}^{\infty }G\left(n\right)\frac{{\left(c_{m}V\right)}^{n}}{n!}e^{-c_{m}V}. \end{array}$$ (4)

The mathematical foundations of this formulation are explained in the S1 File, Section 2.2.1. The hybrid model avoids the use of the interpolation function G, which is highly arbitrary when the originally discrete-valued function *G* cannot be interpolated linearly. Instead, it uses $\bar{G}$, a locally averaged translation rate that results from a Poisson distributed mRNA level, whose mean *c*_*m*_*V* dynamically changes according to the first differential equation. Here, the Poisson distribution serves as an approximation of the real local mRNA distribution. The quality of this approach is discussed later. In order to express $\bar{G}$ in terms of copy numbers, set $\bar{G}(m)≔\bar{G}(\frac{m}{V})\cdot V=\sum_{n=0}^{\infty }G(n)\frac{m^{n}}{n!}e^{-m}\;\forall m\in {\mathbb{R}}_{\geq 0}$.

### Estimation of local Fano factors with the classical and hybrid linear noise approximation

We use local estimates of the Fano factors η*(*M*) and η*(*S*) (variances of mRNA and protein scaled by their means) in order to characterize the relative variability of fluctuations around every stable expression state. The local mean values are approximated by the stable steady states (m*, s*) of a corresponding deterministic model. Local variances are then obtained by LNA, which performs a linearization of all reaction propensities around these fixed points (see SI, Section 1.3 for the theoretical background). For the underlying deterministic model, classical rate laws are normally used, but we use the hybrid deterministic model as a basis for LNA as well. We call the latter approach the *hybrid LNA* (hLNA).

The Fano factor allows for a comparison of the regarded stochastic process (here: mRNA and protein dynamics) to Poisson processes, where *η* = 1 holds [28]. A process is called super-Poissonian if *η* > 1, and sub-Poissonian if *η* < 1. For mRNA fluctuations, the Fano factor is a rather “natural” measure: Without feedback, mRNA formation and degradation events yield a typical birth-death process that follows a Poisson statistic. All deviations from η*(*M*) = 1 can therefore be attributed to transcriptional regulation, independently of the steady-state mRNA level (which is not that simple in terms of the coefficient of variation, an alternative variability measure). Concerning the protein distribution, deviations from *η*(*S*) = 1 show the impact of noise propagation from the mRNA to the protein level, when a system without feedback is considered.

### Characterization of bursts in the protein time course

#### Definition and calculation of burst characteristics

In order to characterize the temporal structure of protein fluctuations, we investigate the occurrence of protein bursts. We use two quantities, namely the *burst size* *α* and the *burst frequency* *ω*. The burst size is generally defined as the mean number of proteins that are translated from a single mRNA molecule [29]. When *G* is nonlinear, the burst size depends on the current state of the system, and we define it as:

$$\begin{array}{c} \alpha \left(m\right)≔\sum_{n=0}^{\infty }\frac{G(n+1)}{n+1}\;\nu \;\frac{m^{n}}{n!}\;e^{-m}\;=\;\frac{\bar{G}\left(m\right)\;\nu }{m} \end{array}$$ (5)

where *m* is the average value around which the mRNA copy number has fluctuated immediately before the burst. In this definition, it is again assumed that the mRNA copy number is approximately Poisson distributed, like in the formulation of the hybrid deterministic model. The derivation of the formula is given in the S1 File, Section 2.5.1.

The burst frequency is the mean number of bursts (i.e., of transcription events) during the lifetime of a protein. When a transcriptional feedback mechanism exists, the frequency depends on the protein level, and we define

$$\begin{array}{c} \omega \left(s\right)≔\frac{F(s)}{\nu }, \end{array}$$ (6)

where *s* is the current mean protein level (see S1 File, Section 2.5.2). *α*(*m*) and *ω*(*s*) are generalizations of the expressions formulated in [28, 29], where only systems without feedback and with linear propensities are studied.

Next, let us determine the average burst characteristics in steady-state. Let (m*, s*) be a stable fixed point of the hybrid deterministic model in terms of copy numbers. Since $\bar{G}(m^{*})=s^{*}$ and *F*(s*) = m* holds, the following relations for the local steady-state burst characteristics α* ≔ *α*(m*) and ω* ≔ *ω*(s*) are obtained:

$$\begin{array}{c} \alpha^{*}=\frac{s^{*}}{m^{*}}\cdot \nu =r\cdot \nu ,\;\omega^{*}=\frac{m^{*}}{\nu }=\frac{s^{*}}{r\cdot \nu }. \end{array}$$ (7)

Here, $r=\frac{s^{*}}{m^{*}}$ denotes the stationary ratio of the protein to mRNA copy number. According to formula (7), bursts in systems with a pre-defined value of s* can be fully characterized in terms of the quantities *r* and *ν*. In our analyses, we usually compare systems with a fixed protein level, so that we can concentrate on the determination of the burst size. The burst frequency then follows directly from the relation s* = α* ⋅ ω*, which means that if s* is kept constant, any change of α* is accompanied by an inverse change of ω*.

#### Graphical characterization of the burst size

The emergence of bursts can be related to differences in the average reaction propensities on the mRNA and protein level: The propensity of the *j*-th reaction *w*_*j*_(*m*, *s*) is defined as the probability per infinitesimal time unit for the *j*-th reaction to occur, depending on the current system state (*m*, *s*) (see Section 1.2 in the S1 File). In steady state, the average propensities of transcription and mRNA degradation, E[F(S)] and E[M], are identical. We denote them by *w*_*m*_, since they are the propensities determining the mRNA dynamics. The average propensities of translation and protein degradation E[G(M)⋅ν] and E[S⋅ν] are identical as well and denoted by *w*_*s*_. Let m* and s* again be the stable steady states of the hybrid deterministic model. Then, *w*_*m*_ ≈ *F*(s*) = m* and $w_{s}\approx \bar{G}(m^{*})\cdot \nu =s^{*}\cdot \nu$. These approximations are based on the same assumptions as the hybrid deterministic model. Since $\bar{G}$ is invertible, we obtain

$$\begin{array}{c} w_{m}=F(\frac{w_{s}}{\nu }),\;w_{m}={\bar{G}}^{-1}(\frac{w_{s}}{\nu }). \end{array}$$ (8)

A connection between propensities and bursts is given by the relation

$$\begin{array}{c} \alpha^{*}\;=r\cdot \nu \;=\;s^{*}/m^{*}\cdot \nu \;=\;w_{s}/w_{m}. \end{array}$$ (9)

This fact illustrates that significant bursts emerge when reactions on the protein level are on average more frequent than reactions on the mRNA level.

The steady state propensities and the burst size α* can be graphically determined by visualizing the two equations in (8) in a *w*_*s*_-*w*_*m*_-plot, and by reading the values at the intersection points of the two graphs, cf. Fig B (panel B) in S1 File. The plot helps in visualizing the effect of the shapes of *F* and $\bar{G}$ and of the interplay of time-scales on the burst size.

### Stochastic model reduction and determination of modes in the protein distribution

Here, we show under which conditions and how the mRNA dynamics can be eliminated from the model: If *ν* ≪ 1, mRNA half-life is extremely short on a time-scale determined by protein degradation. All translation events belonging to one burst thus occur in an infinitesimal timeframe. In the course of model simplification, they can be lumped into a single gene expression event, where multiple protein molecules are formed according to the stochastic distribution of the burst size. This kind of model reduction has been done before, e.g. in [30, 31], though only with fixed burst sizes. In the S1 File Section 2.6, model reduction is performed for state-dependent burst characteristics. The obtained general formulation of the reduced model reads:

$$\begin{array}{lll} \text{reaction}\;1:\; & \emptyset & \overset{F\left(s\right)}{\to }\;B\cdot \text{Protein,}\\ \text{reaction}\;2:\; & \text{Protein} & \overset{\nu }{\to }\;\emptyset , \end{array}$$ (10)

where *B* is the stochastic magnitude of the burst that approximately follows a geometric distribution with mean burst size *α*(*F*(*s*)):

$$\begin{array}{c} P\left(B=b\right)={\left(\frac{\alpha \left(F\left(s\right)\right)}{1+\alpha \left(F\left(s\right)\right)}\right)}^{b}\frac{1}{1+\alpha \left(F\left(s\right)\right)}≕{\text{Geo}}_{\alpha \left(F\left(s\right)\right)}\left(b\right). \end{array}$$ (11)

The reaction scheme is visualized in Fig 1B. The reduction to a one-species-model allows the location of the modes (maxima) in the probability mass function of the protein, similarly to the procedure shown in [32]. As demonstrated in the S1 File Section 2.6.3, all positive-valued modes *s* approximately obey the condition

$$\begin{array}{lll} s=\left\lceil \sigma \right\rceil & \text{where}\;\sigma \in \mathbb{R}, & \sigma +\alpha \left(F\left(\sigma \right)\right)+1=\frac{\alpha \left(F\left(\sigma \right)\right)F\left(\sigma \right)}{\nu }=\bar{G}\left(F\left(\sigma \right)\right). \end{array}$$ (12)

Here, ⌈.⌉ is the ceiling function. The modes can be determined graphically by plotting both sides of the equation in (12) as functions of *σ*, cf. Fig B (panel C) in S1 File.

## Results

In the following, we first evaluate the reliability of the novel approaches used for noise characterization. Then, we present the results of our analyses showing the connection between circuit properties and the variance and structure of fluctuations. We also investigate the relation between the Fano factor and the burst characteristics. Finally, we verify our considerations with stochastic simulations.

### Comparing the results and qualities of the classical and hybrid linear noise approximation

If *G* is linear, i.e. *G*(*m*) = *g* ⋅ *m*, the two deterministic models (3) and (4) and the corresponding two LNA approaches are equivalent since $G(c_{m})=g\cdot c_{m}=g\;\frac{1}{V}\;\sum_{n=0}^{\infty }n\;\frac{(c_{m}\;V{)}^{n}}{n!}\;e^{-c_{m}\;V}=\bar{G}(c_{m})\;\forall \;c_{m}\in {\mathbb{R}}_{\geq 0}.$

Hence, all further discussions on quality differences are only relevant in systems with a translational propensity that depends on mRNA in a nonlinear fashion.

#### Estimation of local mean values

The local mean values of the mRNA and protein probability mass functions are approximated by the stable fixed points m* and s* of the deterministic models. They obey m* = *F*(s*) and

$$\begin{array}{ll} s^{*}=G\left(F\left(s^{*}\right)\right) & \text{in}\;\text{case}\;\text{of}\;\text{model}\;(3)\;\text{and} \end{array}$$ (13)

$$\begin{array}{l} s^{*}=\bar{G}\left(F\left(s^{*}\right)\right)=\sum_{n=0}^{\infty }G\left(n\right)\frac{{\left(F\left(s^{*}\right)\right)}^{n}}{n!}e^{-F\left(s^{*}\right)}\;\text{in}\;\text{case}\;\text{of}\;\text{the}\;\text{hybrid}\;\text{model}\;(4). \end{array}$$ (14)

It can be easily shown that the hybrid deterministic model clearly outperforms the classical rate laws in a system without feedback: In this case, *F* ≡ *a* is a constant function, the mRNA copy number in steady state is indeed Poisson distributed with mean *a* (see SI, Section 2.2.1), and the steady state s* of the hybrid model coincides perfectly with the stochastic mean of the master Eq (2):

$$\begin{array}{c} E\left[S\right]\;=\;E\left[G\left(M\right)\right]\;=\;\sum_{n=0}^{\infty }G\left(n\right)\;\frac{a^{n}}{n!}\;e^{-a}\;=\;\bar{G}\left(a\right)\;=\;s^{*}. \end{array}$$ (15)

In contrast, the steady state of the model based on rate equations is given by s* = *G*(*a*), i.e. by a point evaluation of an (arbitrary!) interpolation of the originally discrete function *G*. If *G* is nonlinear, *G*(*a*) usually differs from $\bar{G}(a)$ (cf. Fig A in S1 File) and is thus a worse estimate of the stochastic mean.

To be more general, the error of the hybrid deterministic model is expected to become very small when the transcription rate *F* is almost constant in the range of local protein fluctuations, since the local mRNA distribution is then well approximated by a Poisson distribution. For systems with more sensitive feedback, the quality of the hybrid ansatz might be reduced. Nevertheless, we have observed that it still outperforms the classical model due to the local averaging of *G*, which yields a more realistic estimate of the effective (deterministic) translation rate (see the following section, as well as Section 3.1 in the S1 File, where we have evaluated several exemplary reaction systems).

#### Estimation of local Fano factors

The calculations of the local Fano factors η*(*M*) and η*(*S*) around a stable fixed point (m*, s*) are carried out in detail in the S1 File, Section 2.3. The obtained formula read:

$$\eta^{*}\left(M\right)=1+\frac{f}{g^{-1}-f}\left(\frac{\nu }{1+\nu }+\frac{1}{1+\nu }\cdot \;r\frac{f}{g}\right)$$ (16)

$$\begin{array}{ll} \eta^{*}\left(S\right)=1+\frac{f}{g^{-1}-f}\left(\frac{1}{1+\nu }+\frac{\nu }{1+\nu }\cdot \frac{1}{r\frac{f}{g}}\right) & \text{if}\;f\neq 0\text{,} \end{array}$$ (17)

$$\eta^{*}\left(S\right)=1+\frac{\nu }{1+\nu }\frac{g^{2}}{r}\;\text{if}\;f=0.$$ (18)

Here, $f≔\frac{d\;ℱ(c_{s})}{d\;c_{s}}{|}_{c_{s}=c_{s}^{*}}$ and

$$\begin{array}{ll} g≔\frac{dG\left(c_{m}\right)}{dc_{m}}{|}_{c_{m}=c_{m}^{*}} & \text{in}\;\text{case}\;\text{of}\;\text{model}\;\left(3\right), \end{array}$$ (19)

$$\begin{array}{l} g≔\frac{d\bar{G}\left(c_{m}\right)}{dc_{m}}{|}_{c_{m}=c_{m}^{*}}=\sum_{n=0}^{\infty }\left(G\left(n+1\right)-G\left(n\right)\right)\frac{{\left(m^{*}\right)}^{n}}{n!}e^{-m^{*}}\;\text{in}\;\text{case}\;\text{of}\;\text{model}\;\left(4\right). \end{array}$$ (20)

According to these formula, the Fano factors depend on a local derivative of G if the classical LNA is used. The derivative can vary considerably with the chosen interpolating function and is therefore quite arbitrary. In contrast, if the novel hLNA approach is applied, *g* is an average difference quotient (Eq (20)) that only uses the original discrete function values of *G*. Besides the values of *g*, the values of *r* and *f* might differ between the two LNA approaches, since the fixed points of the underlying deterministic models are not necessarily identical, as we have just seen.

**Fig 2:**
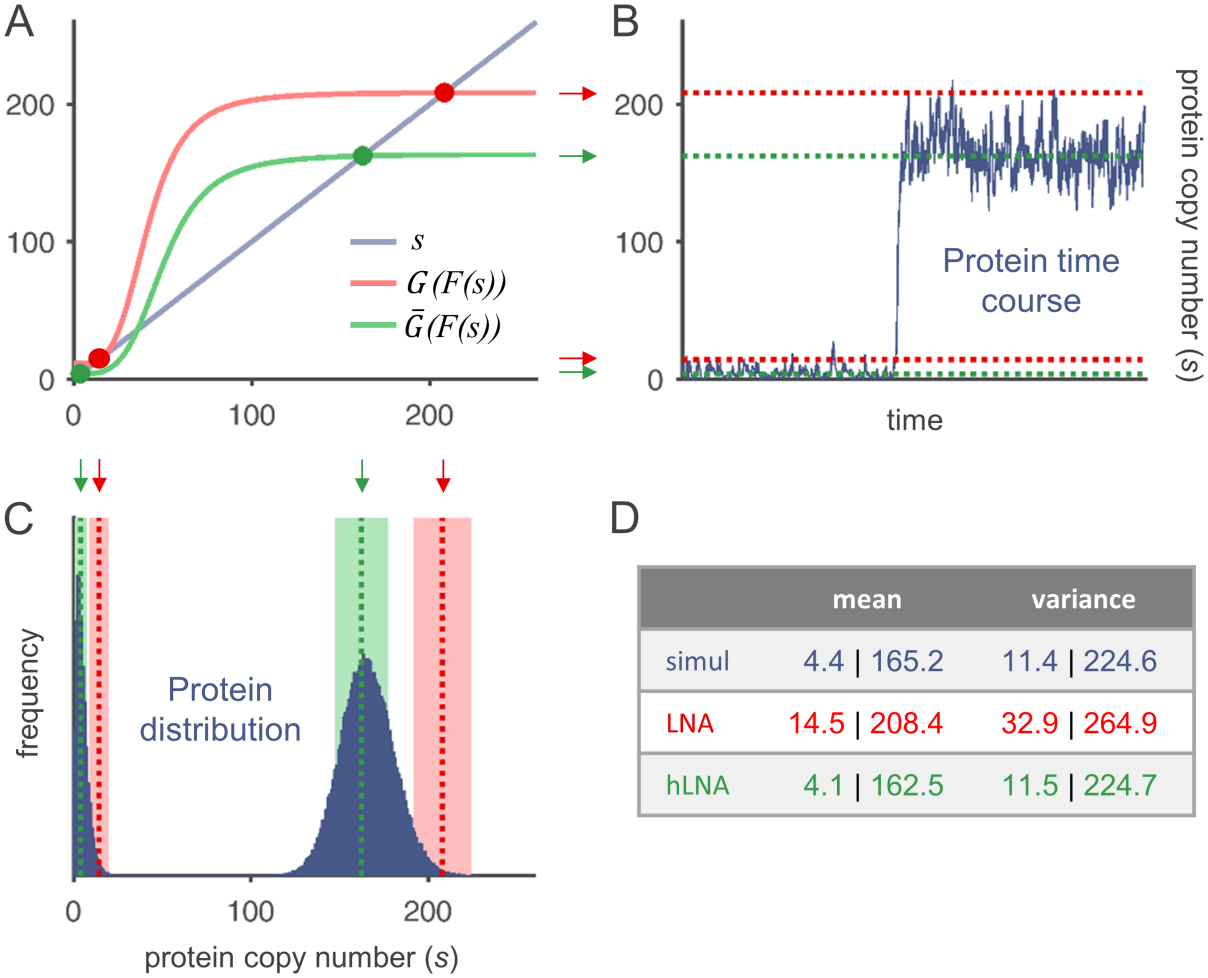
Quality of the classical and hybrid linear noise approximation. (A): Graphical determination of stable fixed points according to Eqs (13) and (14). The intersection points marked in red and green correspond to the stable fixed points of the classical and hybrid deterministic model, respectively. (B): Simulated protein time course with a transition from the inactive to the active expression state. The locations of the stable fixed points according to (A) are indicated by dashed lines. (C): Protein distribution (histogram) of the simulated time course. In addition to the estimated local means, the variance obtained by classical and hybrid LNA is visualized by colored areas (mean ± standard deviation). (D): Quantitative comparison of estimates with values extracted from the simulation. Functions and parameters: $F(s)=0.022+1.46\;\frac{s^{4}}{s^{4}+58^{4}}$, $G(m)=281\;\frac{m}{m+0.51}$, *ν* = 0.01.

What about the quality of the hLNA? Fig 2 shows a simulated protein trajectory and the corresponding protein distribution of a representative bistable circuit with nonlinear *G*. The local mean values and variances extracted from those simulated data are compared with the estimates based on the classical and hybrid LNA. Obviously, the simulated local means correspond well to the stable fixed points of the hybrid deterministic model, while large deviations occur when the classical deterministic model is used. Reliable estimates of local mean values are crucial for the quality of the linear noise approximation, since they are the points around which linearization is performed. As expected, the local variances obtained from hLNA are much better estimates than those calculated with classical LNA. This observation is corroborated by the analysis of further exemplary reaction systems in Fig C in S1 File. Therefore, from now on, the novel hLNA approach is used for all analyses and discussions of local means and Fano factors.

From a protein-mRNA phase plot of the hybrid deterministic model, most of the decisive circuit properties can be read (cf. Fig B (panel A) in S1 File). Therefore, such a plot can—at least qualitatively—support the construction of circuits with certain noise patterns.

### Quality of the reduced model and of the determination of modes

As already mentioned, the condition *ν* ≪ 1 is crucial for model reduction, since otherwise, the translation events could not be condensed into a single reaction. However, the quality of the reduced model depends on further conditions:

An excellent quality is assured when *G*, the translation propensity, is linear: Under this condition, each transcription event does indeed lead to a geometrically distributed burst with mean α*, independently of the current mRNA copy number. The mRNA level can thus be easily eliminated from the model.

If *G* is nonlinear, the mRNA level has an impact on the burst size and can thus only be eliminated if a reasonable assumption about its distribution can be made based on the current protein level. We have assumed that between bursts, the mRNA distribution is Poisson with a mean *F*(*s*) that steadily adapts to the current protein copy number (pseudo-steady-state assuption), but that the distribution does not change during a burst; under this condition, burst sizes are again geometrically distributed with a dynamic mean *α*(*F*(*s*)). One criterion that fulfills this condition is a transcriptional propensity *F* that is constant in the region of protein fluctuations.

If none of the criteria, linear *G* and constant *F*, is fulfilled, the average magnitude of bursts might systematically be over- or underestimated, leading to a reduced model whose protein distribution has too broad or too narrow peaks. However, a number of simulations showed that mode estimation is still robust, cf. the S1 File, Section 3.3.

### Interpreting the influence of circuit properties on Fano factors

From this section onward, we will interpret the obtained formula from a biological point of view. First of all, the impact of circuit properties on the magnitude of noise will be examined based on Eqs (16)–(18). The Fano factors depend on the four parameters *f*, *g*, *r*, and *ν*: *f* and *g* can be interpreted as the local sensitivity of the transcription and translation rate to fluctuations in the protein and mRNA level, respectively. Besides that, the protein-to-mRNA ratio *r* and the relative time-scale of protein dynamics *ν* play a role. Note that if *G* is linear, i.e. *G*(*m*) = *g* ⋅ *m*, the relation *r* = *g* holds.

**Fig 3:**
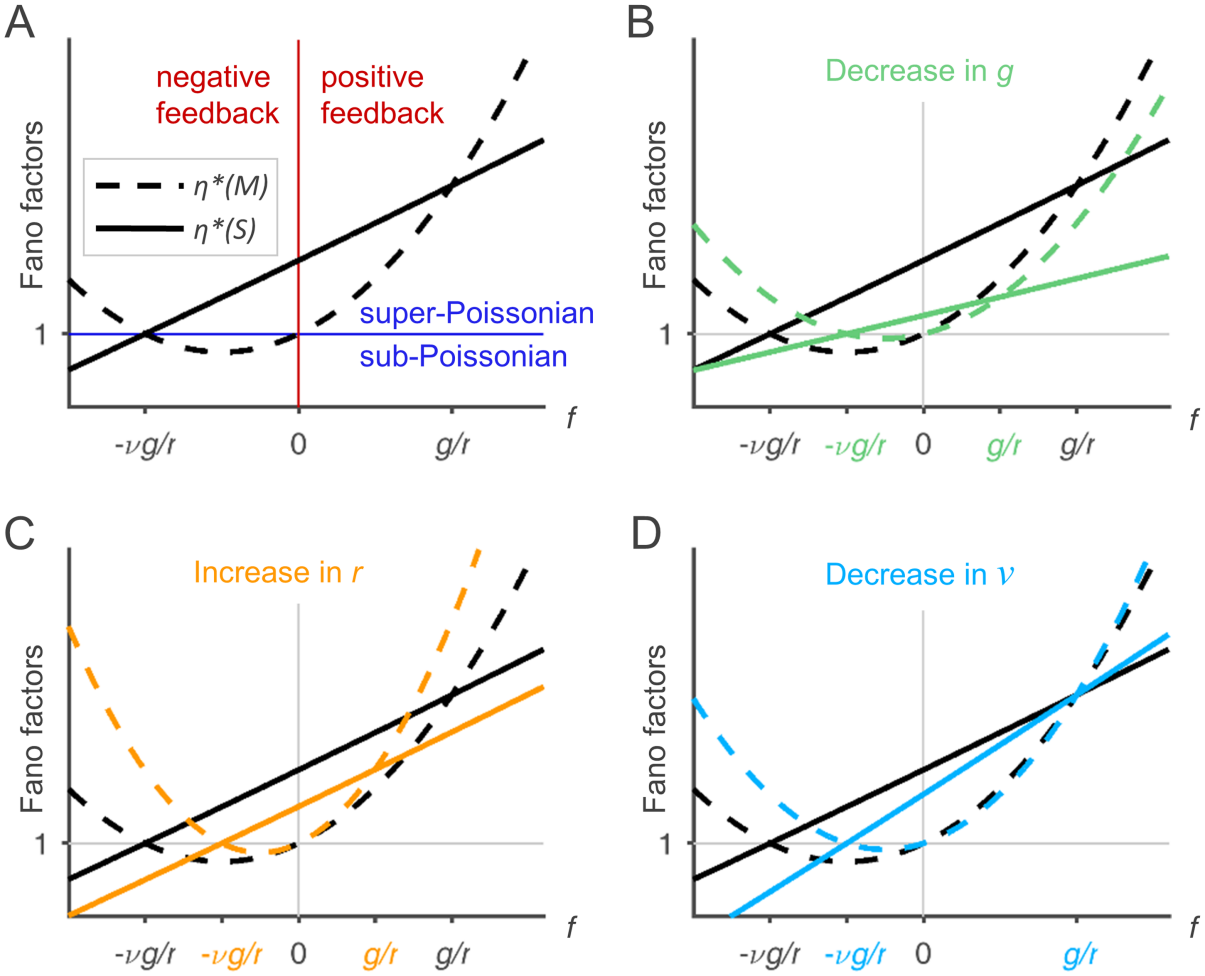
Results of the linear noise approximation. The plots show the dependence of η*(*M*) (dashed line) and η*(*S*) (solid line) on *f* with fixed values of *g*, *r*, and *ν*. In panels (B) to (D), parameters *g*, *r* and *ν* are varied (colored lines) in order to illustrate their effect on the noise of the system. The parameters are as follows: (A): *g* = 1, *r* = 100, *ν* = 1; (B): *g* is halved compared to (A), i.e. *g* = 0.5, *r* = 100, *ν* = 1; (C): *r* is doubled compared to (A), i.e. *g* = 1, *r* = 200, *ν* = 1; (D): *ν* is halved compared to (A), i.e. *g* = 1, *r* = 100, *ν* = 0.5.

We start by considering the effect of *f*, which fundamentally characterizes the system by defining the mode of autoregulation. In case of missing local feedback (*f* = 0), the distribution of *M* in the linearized system is Poissonian (i.e. η*(*M*) = 1), which is in line with the expectations, since under these conditions, mRNA dynamics follow a standard birth-death-process. Subsequent noise propagation from *M* to *S* leads to a super-Poissonian protein distribution (η*(*S*) > 1). With increasing positive feedback strength (*f* > 0), noise is amplified until finally, η*(*M*) and η*(*S*) diverge to infinity for *f* → *g*^−1^. Note that *f* < *g*^−1^ holds in any case, since otherwise, the fixed point (m*, s*) would not be stable (see S1 File, Section 2.2.2). In contrast, negative autoregulation (*f* < 0) leads to protein noise attenuation. Repression can be strong enough to create sub-Poissonian protein distributions (η*(*S*) < 1) with $\frac{\nu }{1+\nu }$ as a lower bound. The effect of autorepression on mRNA noise is slightly more complex: for decreasing *f* < 0, mRNA noise first decreases to sub-Poissonian levels but then increases again, indicating over-regulation. This shows that efficient protein noise attenuation can be achieved through negative feedback at the expense of enlarged mRNA fluctuations. In Fig 3A, η*(*M*) and η*(*S*) are illustrated as functions of *f*. From the graphs, one can read the regions where the fluctuations are super- and sub-Poissonian, respectively. η*(*M*) = η*(*S*) holds if either $r\frac{f}{g}=1$ or $r\frac{f}{g}=-\nu$.

The effect of a decrease in the translational sensitivity *g* is as follows: In case of positive feedback, it reduces noise propagation through the circuit and therefore leads to smaller mRNA and protein fluctuations. Under negative feedback, η*(*M*) is amplified. η*(*S*) is increased if the local negative feedback is strong ($f<-\frac{2\nu g}{r-\nu g^{2}}$) and if $\frac{\nu \;g^{2}}{r}<1$, otherwise reduced. The effects are illustrated by the colored lines in Fig 3B.

Increasing the protein-to-mRNA ratio *r* reduces protein fluctuations, but augments mRNA fluctuations (cf. Fig 3C), unless *G* is linear. In this case, a change in *r* is coupled to a change in *g*. An increase in *r* = *g* then leads to an increase in η*(*S*) if *f* > −*ν*, and to an increase in η*(*M*) if *f* > 0 or if *f* < −*ν*. Otherwise, the Fano factors are reduced.

Concerning the time scales of mRNA and protein dynamics, the formula show that slowed protein dynamics (*ν* ↓) lead to protein noise attenuation if $r\frac{f}{g}<1$, but promote protein fluctuations if $r\frac{f}{g}>1$. mRNA noise is diminished if $0<r\frac{f}{g}<1$ and enhanced otherwise (cf. Fig 3D). The expression $r\frac{f}{g}$ can be interpreted as the ratio of capabilities to transmit noise from mRNA to protein and back from protein to mRNA. Under positive feedback, the system gets less noisy if the reaction with the predominant capability of noise propagation is slow, so that noise is partially averaged out.

All in all, it can be stated that a strong influence of mRNA fluctuations on protein dynamics (high *g*, low *r*) and high positive feedback strength (high *f*) intensify protein noise. Sub-Poissonian protein distributions can only be achieved through negative feedback. The effects can be supported by an appropriate choice of *ν*.

### Influence of bursts

#### Peaks in the protein time course

By looking at the burst measures α* and ω*, we can better characterize the temporal structure of protein noise. To do so, we first like to illustrate and interpret the measures and thereby get a more intuitive understanding of them:

When the mRNA half-life is short (*ν* ≪ 1) and the burst frequency ω* is low, the single burst events generate distinct peaks in the protein time course. Under these conditions, the measures can be read directly from the shape of mRNA and protein fluctuations over time. The procedure is developed and explained in the S1 File, Section 4.1; here, we only list the most important facts: The burst size α* corresponds approximately to the average height of protein peaks β*, i.e. to the amount by which the protein level increases due to a burst. To be more precise, α* = β* ⋅ (1 + *ν*). The factor 1+ *ν* takes account of the amount of proteins that are instantaneously degraded during the burst. It can usually be neglected since mRNA half-life is much shorter than the half-life of most proteins. The burst frequency ω* can be estimated from the average temporal distance of the peaks. However, when the mRNA level rises, the measures cannot be extracted directly anymore since the peaks might overlap and single bursts cannot be clearly distinguished. Positive feedback promotes overlaps as well, as will be shown in the next section.

From Eq (7), we can tell by which circuit modifications the burst measures can be varied without affecting the average protein level s*: α* can be raised by increasing the average protein-to-mRNA-ratio *r* or the time-scale parameter *ν*. The burst frequency is then decreased accordingly. An increase in *r* implies an increase in the scaled translation propensity *G* and a simultaneous decrease in the scaled transcriptional activity *F*, so that the mRNA level m* is reduced while s* is kept constant. An enhancement of *ν* is achieved by an acceleration of the protein dynamics (scaled translation and degradation) relative to the mRNA dynamics. Note that although the two kinds of modifications (increase of *r* or *ν*) can have identical effects on α*, the effect on the maximum peak height β* is less pronounced when *ν* is increased due to the enhancement of protein degradation.

#### Links between Fano factors and burst characteristics

In this section, we interpret burst measures and Fano factors in the context of each other. In the easiest case of an unregulated transcription rate (*f* = 0) and a linear translation rate (*G*(*m*) = *g* ⋅ *m* = *r* ⋅ *m*), Eq (18) can be simplified to

$$\begin{array}{c} \eta^{*}\left(S\right)\;=\;1\;+\;\frac{\alpha^{*}}{1\;+\;\nu }\;=\;1\;+\;\beta^{*}. \end{array}$$ (21)

Usually, *ν* is so small that it is negligible, i.e. a direct relation between the protein burst size and the variability of fluctuations exists (cf. [28]). If *f* is nonzero due to transcriptional feedback, we obtain

$$\begin{array}{c} \eta^{*}\left(S\right)\;=\;1\;+\;\frac{\alpha^{*}\;+\;f\cdot r}{\left(1\;-\;f\cdot r)\cdot (1\;+\;\nu \right)}. \end{array}$$ (22)

Obviously, feedback provides large flexibility to adjust η*(*S*) via *f* while keeping α* and ω* constant. In the following, we try to fully express η*(*S*) in terms of “extended” bursting properties: By calculating the local derivatives of the state-dependent burst measures ${\alpha^{\prime }}^{*}≔\frac{d\;\alpha (m)}{d\;m}{|}_{m=m^{*}}=\frac{g\;m^{*}-\bar{G}(m^{*})}{(m^{*}{)}^{2}}\nu =\frac{g}{\omega^{*}}-\frac{\alpha^{*}}{\omega^{*}\;\nu }$ and ${\omega^{\prime }}^{*}≔\frac{d\;\omega (s)}{d\;s}{|}_{s=s^{*}}=\frac{f}{\nu }$ (here, Eqs (5) and (6) are differentiated and Eq (7) is used), one obtains

$$\begin{array}{c} f\;=\;{\omega^{\prime }}^{*}\cdot \nu \;\text{and}\;g\;=\;\alpha^{*}/\nu \;+\;{\alpha^{\prime }}^{*}\cdot \omega^{*}. \end{array}$$ (23)

**Fig 4:**
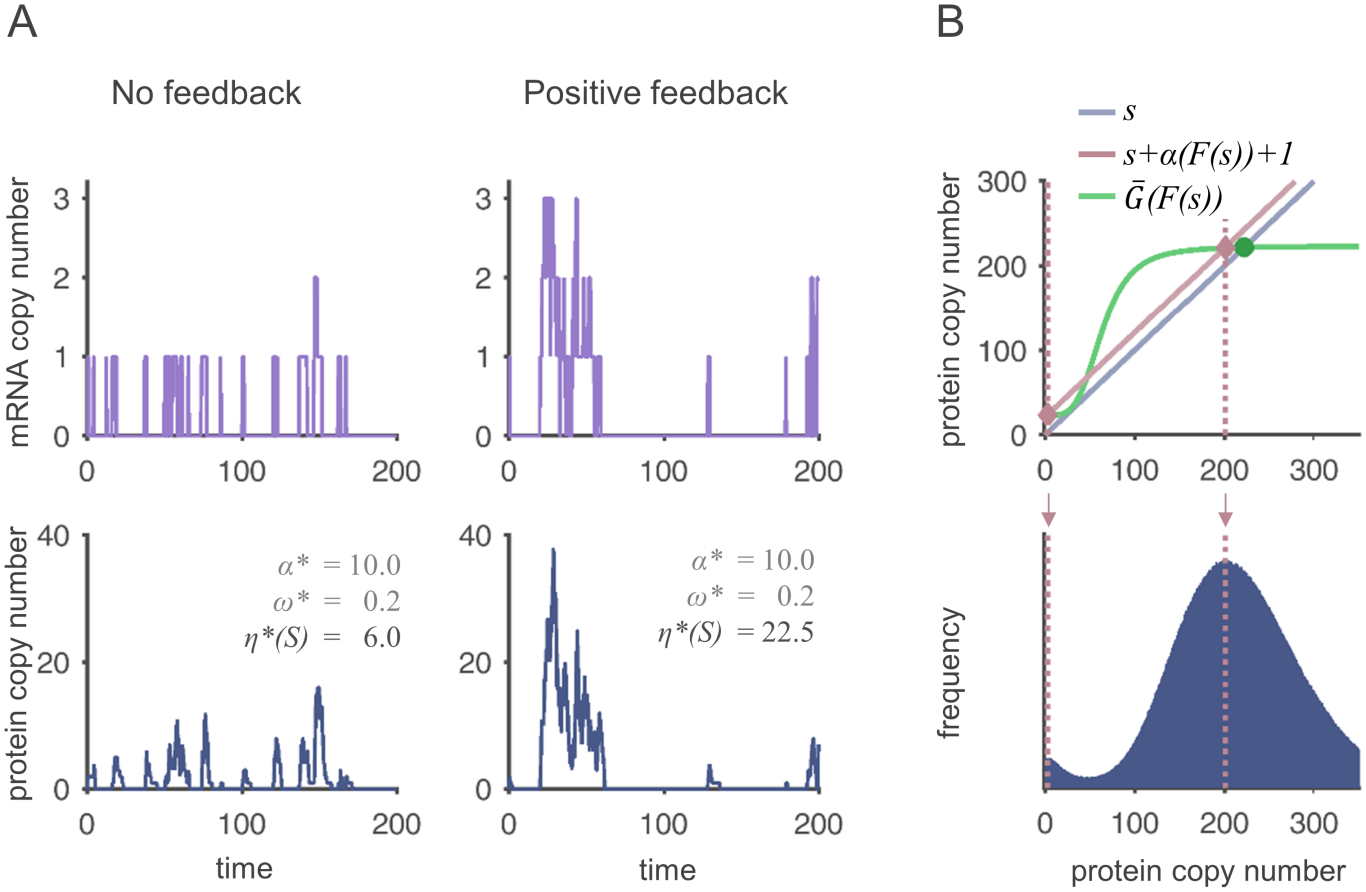
Protein bursts cause peaks in protein time courses and skewed protein distributions. (A): mRNA (upper plots) and corresponding protein time courses (lower plots) of reaction systems without (left) and with (right) positive feedback. The average burst sizes and frequencies are identical in the two systems, but the transcription events are temporally clustered under positive feedback, leading to rare super-bursts. The functions used are *F*(*s*) = *a* + *v* ⋅ *s* and *G*(*m*) = *g* ⋅ *m* with *a* = 0.2 and *v* = 0 in the system without feedback and *a* = 0.05 and *v* = 0.075 in the system with feedback. In both cases, *g* = 10 and *ν* = 1. (B): The upper plot shows the graphical determination of the location of modes. Two modes are predicted in the monostable system (modes: rose diamonds, fixed point: green filled circles). The protein distribution in the lower figure shows that the prediction is exact. Here, $F(s)=7/600+0.1\cdot \frac{s^{4}}{s^{4}+64^{4}}$, *G*(*m*) = 2000 ⋅ *m*, *ν* = 0.01.

The first relation in Eq (23) shows that the presence of feedback leads to a state-dependent burst frequency (*ω*′* ≠ 0), so that the bursts are inhomogenously distributed over time. Positive feedback, for example, promotes the temporal clustering of bursts, as can be seen in Fig 4A: The increase in the protein level during a burst stimulates further bursts, while bursts are rare when the protein level is low. Overlapping bursts appear as rare “super-bursts” with large amplitudes. From the previous analysis of the impact of *f*, we can tell that this enhances η*(*S*).

The second equation in (23) shows that the translational sensitivity *g* is determined by the burst size in and around the steady state (α* and *α*′*). The burst size is state-dependent if *G* is nonlinear. For example, if *G* is concave, *α*′* < 0, i.e. an increase in mRNA is accompanied by a reduction of protein peaks heights. Compared to an almost identical system with same α*, but with linear or convex *G*, the translational sensitivity is therefore reduced.

By replacing *f* and *g* in Eq (17) with the above expressions, one sees how the inhomogeneities affect the Fano factor:

$$\begin{array}{c} \eta^{*}\left(S\right)\;=\;1\;+\;\frac{\left({\alpha^{\prime }}^{*}\cdot \omega^{*}\cdot \nu \;+\;\alpha^{*}\right)\cdot \left({\alpha^{\prime }}^{*}\cdot \omega^{*}\cdot \nu \;+\;\alpha^{*}\cdot \left(1\;+\;{\omega^{\prime }}^{*}\right)\right)}{\alpha^{*}\cdot \left(1\;+\;\nu \right)\cdot \left(1\;-\;{\omega^{\prime }}^{*}\cdot \left({\alpha^{\prime }}^{*}\cdot \omega^{*}\cdot \nu \;+\;\alpha^{*}\right)\right)}. \end{array}$$ (24)

This formula finally captures the connection between η*(*S*) and protein fluctuation patterns. In contrast, Eq (17) is better suited for relating the Fano factor to circuit properties. Note that *ν* (or, after reformulation, *r*) cannot be eliminated from the above expression, i.e. η*(*S*) still contains information which is not covered by *α*(*m*) and *ω*(*s*). Neither can the burst characteristics be estimated based on the sole knowledge of η*(*S*). This demonstrates that both the burst size and the Fano factor contain relevant information on characteristics of protein fluctuations.

#### Links between the skewness of the protein distribution and bursts

A comparison of the modes (Eq (12)) with the local mean values s*, which obey $s^{*}=\bar{G}(F(s^{*}))$, shows the influence of the burst size on the skewness of the protein distribution: For very small bursts, the mode corresponds approximately to the mean (like in a standard Poisson distribution). When the burst size is increased, the mode is shifted to the left (right-skewness). This makes sense as the burst properties determine whether the protein level constantly fluctuates around the mean value (small α*, large ω*), or whether it is mostly below average with rare peaks (large α*, small ω*).

Fig 4B illustrates the graphical determination of fixed points and modes and compares the estimates with simulated distributions. Obviously, the prediction is very good, although in this system, skewness is so pronounced that despite the system being monostable, a second mode arises at low protein levels.

### Application: Modulating the robustness of fixed points in a bistable feedback system

Our results are now used to design bimodal regulatory circuits with specific noise patterns. The noise level of a protein expression state is associated with its robustness, as large fluctuations facilitate random transitions to the other state. However, Fano factors and burst characteristics are insufficient for quantitatively predicting transition probabilities (e.g., in terms of mean first passage times). They rather serve as qualitative measures of robustness with which analogous circuits can be compared.

#### System with linear translation rate

For a start, a bistable system with a linear translation propensity *G* is regarded. Bistability is achieved by a sigmoid shape of the transcription propensity *F*, which commonly emerges from cooperative feedback. The general formula are: $F(s)\;=\;a+v\;\frac{s^{n}}{K_{s}^{n}+s^{n}}$, where *a* and *a*+ *v* are the basal and maximum transcription propensities, respectively, *K*_*s*_ is the microscopic dissociation constant and *n* is the Hill coefficient, and *G*(*m*) = *g* ⋅ *m*.

Since *g* = *r*, the local Fano factors can only be adjusted by three parameters, *ν*, *g*, and *f*. The parameters *ν* and *g* are identical at both stable expression states and therefore only suitable for adjusting the overall noise strength in the system. The burst size α* = *g* ⋅ *ν* is constant throughout the system as well. A decoupling of the noise levels at the two states can thus only be achieved by a difference in *f*.

For generating a unidirectional activating switch that is e.g. suitable for competence regulation, the feedback function *F* should be saturated in the competent state, while it is sensitive to protein fluctuations in the inactive state, thereby promoting superbursts.

#### Systems with nonlinear translation rate

Much larger flexibility is reached when the translation propensity is nonlinear. Here, we demonstrate this fact based on the function $G(m)=u\;\frac{m}{K_{m}+m}$, a typical Michaelis-Menten function. As before, *ν* is only suitable for regulating overall noise. However, as *G* depends on two parameters, the degrees of freedom that are available to adapt noise are increased. *g* and *r* are neither necessarily identical nor constant throughout the system anymore. Let us now check whether the given shape of *G* promotes a unidirectional switch.

First, we qualitatively compare the Fano factors at the two stable steady states $\left(m_{1}^{*},s_{1}^{*})<(m_{2}^{*},s_{2}^{*}\right)$. One can show that if *G* is (strictly) concave, $\bar{G}$ is (strictly) concave as well (S1 File, Section 4.2). As a consequence, *g* decreases with increasing m*. Moreover, also the quotient $\frac{g}{r}$ is smaller in the high expression state. From Eq (17) we can tell that this supports the required noise pattern, i.e., that $\eta_{1}^{*}(S)>\eta_{2}^{*}(S)$. Moreover, due to the concavity of $\bar{G}$, $\alpha_{1}^{*}>\alpha_{2}^{*}$. That means that in the inactive state, rare but large protein peaks can lead to a random stimulation of some cells. In the active state, peaks have reduced amplitudes and fluctuations are more symmetrical.

A visual illustration of these results can be found in Fig B in S1 File, where different kinds of plots show the dependence of the noise measures on circuit properties. In the following section, the analytical considerations are verified by stochastic simulations.

### Stochastic simulations of a bistable system

**Fig 5:**
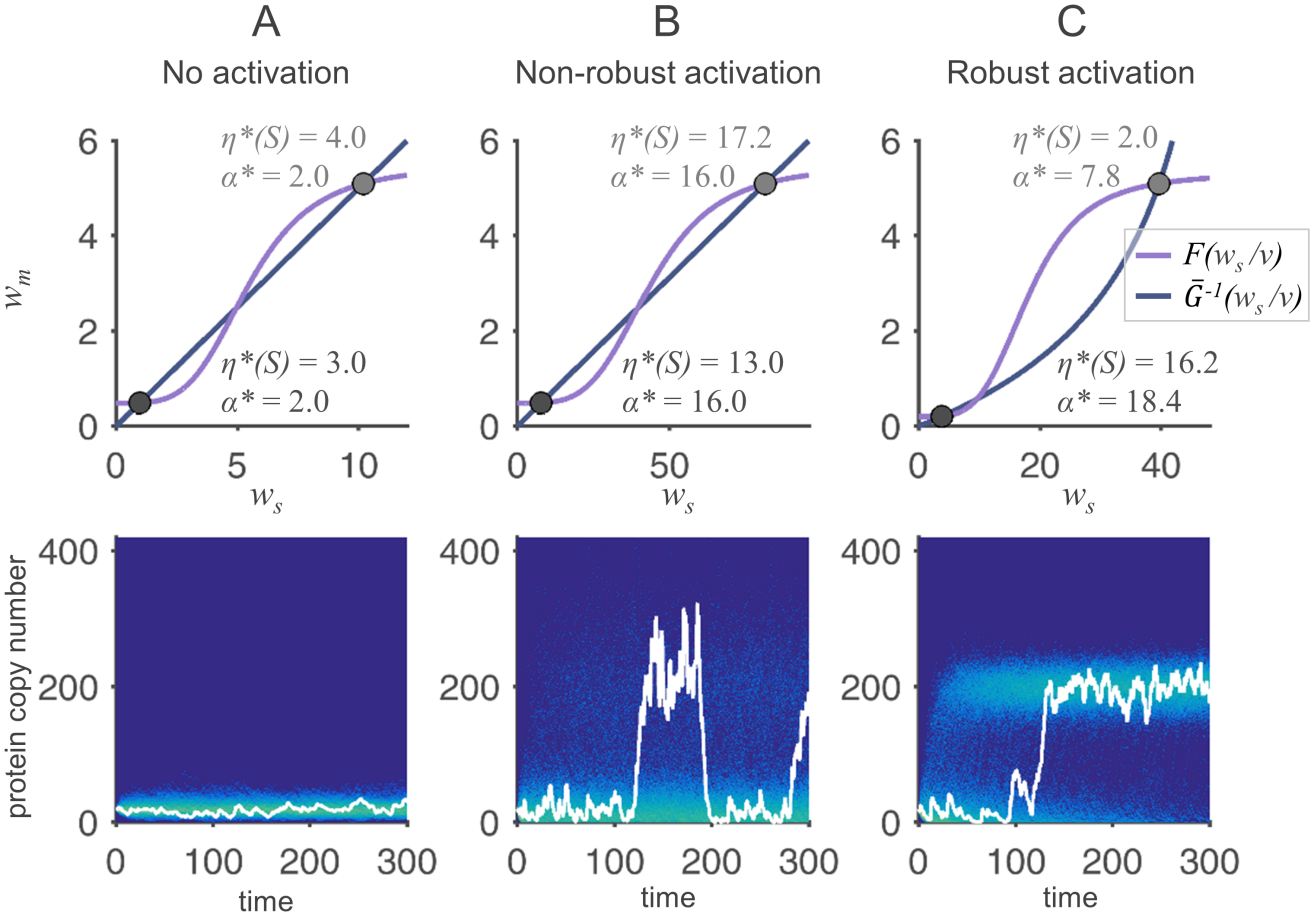
Stochastic simulations of bistable autostimulatory reaction systems. In the upper part, *w*_*s*_-*w*_*m*_-plots of three different regulatory systems with similar protein levels in the active states are shown. The plots in the lower part show the results of 500 simulations of corresponding protein time courses, initialized in the inactive state. The protein distributions are visualized by color plots. One exemplary time course is highlighted in white. (A): Linear translation rate. Protein bursts are too small to activate the system. $F(s)=0.49+5\;\frac{s^{4}}{s^{4}+110^{4}}$, *G*(*m*) = 40*m*, *ν* = 0.05. (B): Bursts emerge through accelerated protein dynamics (increase in *ν*), leading to random activation and inactivation events. Parameters as in (A), except for *ν* = 0.4. (C): System with saturated translation rate. Bursts occur in the inactive state, but are attenuated in the active state, leading to a bimodal, robust activation of the system. $F(s)=0.2+5.12\;\frac{s^{4}}{s^{4}+91^{4}}$, $G(m)=290\;\frac{m}{m+2}$, *ν* = 0.2.

In Fig 5, three different bistable systems with cooperative autostimulation are simulated. In panel (A), the translation rate depends linearly on *m*. Therefore, the value of the burst size α* is equal at both fixed points. Since protein dynamics are chosen to be much slower than mRNA dynamics, the bursts in the inactive state are too small to randomly activate the system. In panel (B), the value of *ν* is increased. As a consequence, the burst sizes α* and the Fano factors η*(*S*) are amplified at both fixed points. Protein bursts in the inactive state now allow for a random activation of the system. However, the active state is likewise destabilized. The system thus switches between the two states. Panel (C) shows that a saturated translation rate leads to significant differences in α* and in η*(*S*) at the two stable steady states. Clear bursts can be observed in the inactive state, leading to an activation of a subpopulation of cells, while protein noise is attenuated in the active state. The active state is therefore highly robust, and the simulated cells show a clearly bimodal protein distribution. For a more quantitative view on the robustness of the states, first passage times are extracted from simulation data. They are discussed in the S1 File, Section 4.3.

Note that in the bistable systems we have just regarded, the local feedback sensitivity *f* is small in both expression states due to the sigmoid shape of *F*. Therefore, we expect the results of hLNA and burst characterization to be reliable.

## Discussion

In this study, we have systematically investigated mRNA and protein noise in a single-gene autoregulatory system. The results are in accordance with previous studies about the impact of simple feedback and of reaction time scales on noise [8–10]. Here, we have put special effort into the analysis of a system that is multimodal, and whose multimodality is related to deterministic multistability. For this kind of multimodal distributions, an analytical and biologically reasonable approach by which they can be unequivocally split into unimodal ones does not exist. By contrast, a multimodal distribution that is generated by a slowly switching component (e.g. promoter state) is often a mixture of few unimodal distributions and can be modeled as such with the help of conditional probabilities [33, 34]. Coming back to our system, the main difficulty is that the “local” measures by which we like to quantify state-specific noise are only hypothetical values. Because of that, the quality of estimates was evaluated by comparing them with values extracted from stochastically simulated time courses, in which distinct expression states could be clearly identified (cf. Fig 2 and Fig C (panel D) in S1 File).

For the quantification of noise, we suggest an analysis of both the local Fano factors and the local burst characteristics for each stable protein expression state. By combining the results, the intensity and shape of fluctuations can be rated. The calculation of local Fano factors was originally based on the linear noise approximation, which yields exact results when all reaction propensities are linear. In the nonlinear case, closed-form approximations could be obtained, but their quality was often poor. Alternative methods had been developed by other groups, for example effective mesoscopic rate equations (EMREs) [35] and the inverse omega square method [36] that incorporate terms of higher order than LNA. For monostable systems, these methods were shown to greatly improve the quality of the estimates. However, they are not applicable to multistable systems. We have therefore developed a novel approach that is inspired by LNA, but in which the original deterministic description is replaced by a novel hybrid-deterministic model. This model takes fluctuations of the discrete mRNA copy number into account and was shown to better describe the average behavior of the reaction system. We like to point out that—in contrast to model simplifications where some stochastic variables are replaced by deterministic ones [30, 31]—we only use the deterministic description for linearizing the fully stochastic model (hybrid LNA). The noise estimates obtained through this approach proved to be much better than the ones obtained through classical LNA. Even though only single-gene systems have been regarded in this work, the approach can be applied to systems involving multiple genes (and, hence, multiple mRNA species) by describing every nonlinear translation reaction by a hybrid deterministic rate. Furthermore, systems with more than two stable expression states can be analyzed as long as the hybrid deterministic model reflects this multimodality. Even systems with fluctuating low-copy-number species other than mRNA could be regarded, but it might be advantageous to replace the Poisson probability mass function by another closed-form distribution.

For the characterization of translational bursts, we have introduced the state-dependent burst size and burst frequency as a generalization of the definitions in [28, 29]. Our expressions are consistent with the formulation of the hybrid-deterministic model, so that connections between the protein Fano factor and the structure of protein bursts could be identified. We have also shown analytically that large bursts may lead to a right-skewness of the distribution. In an extreme case, the connection between multimodality and multistability may be disrupted (as is shown in Fig 4 and discussed extensively in [32]). If this happens, the hLNA method is not capable of characterizing all peaks anymore. However, our method to predict the location of modes still works reliably. By comparing the number and location of modes and stable steady states (according to the hybrid-deterministic model), one can therefore decide a-priori whether the application of the hLNA is appropriate.

Besides the computational analysis, we have proposed methods for studying the noise measures graphically (cf. Fig C in S1 File): A deterministic protein-mRNA-phase-plot can help to predict the variance of fluctuations qualitatively. Furthermore, a *w*_*s*_-*w*_*m*_-plot (plot of propensities) can be used for evaluating the burst size. The location of fixed points and modes can also be determined and compared graphically, giving information on the skewness of the protein distribution. All these plots support an intuitive evaluation of regulatory systems with respect to their noise patterns and are useful tools for the design of synthetic circuits. We can now predict how modulating the strengths of the promoter, feedback, or ribosomal binding site, or an engineering of the mRNA or protein stability changes the noise pattern. However, we need to mention that despite our methods yielding measures of local noise, they can only make qualitative predictions on transition probabilities or population distributions.

As an example, we have found that if the translation propensity is linearly dependent on the mRNA level, the noise levels of the expression states in a single-gene bistable system are coupled. In contrast, nonlinear translation, which might occur due to global effects like cellular resource limitation, leads to an increased flexibility in the interplay of the mRNA and protein dynamics so that various noise patterns can be created. This provides a possible explanation on how certain stochastic decision-making processes can proceed reliably. In *S. mutans*, for example, upregulation of the protein ComS leads to the entry of a cell into genetic competence. Experiments have shown that production of ComS occurs in a bimodal fashion [20], and this was attributed to deterministic bistability caused by a positive feedback loop [37]. However, simple stochastic simulations of systems with linear translation rates like those in Fig 5A and in Fig 5B showed either a lack in responsiveness or uncontrolled switching between ON and OFF states—a fact that remains obscure in deterministic modeling. Upregulation of *comS* expression stimulates the synthesis of numerous downstream gene products. To be functional and economic, the high expression state should persist for a certain time, before it is shut off in a controlled manner. On the other hand, the intense production of all the other proteins might lead to a limitation in ribosomal capacity. This would result in saturation of the translational propensity function *G* at high *comS* mRNA levels. As our analyses have shown, concavity of *G* can indeed circumvent the problem of stochastic deactivation. In an analogous manner, it can be shown that convexity of *G* would destabilize the active state while making the inactive state more robust.

All in all, we have established a mathematical framework for exploring the capability of a minimal autoregulatory circuit to modulate noise. It leaves room for various extensions: For example, promoter state switching and transcriptional bursts can be included. To do so, the above mentioned model in [34] could be used as it is based on LNA as well and therefore compatible to our approach. Furthermore, the dynamics of cooperative feedback could be studied in more detail, instead of describing it by a simple transcriptional function [38, 39].

## Supporting information

S1 File Supporting information PDF file. (PDF)
