# Supplementary material for: Characterization of noise in multistable genetic circuits reveals ways to modulate heterogeneity
Source: PLoS One. 2018 Mar 26;13(3):e0194779. doi: 10.1371/journal.pone.0194779 (PMC5868838; doi:10.1371/journal.pone.0194779)
Supplement: S1 File — (PDF) [file pone.0194779.s001.pdf]

Supporting Information: Characterization of noise in multistable genetic circuits reveals ways to modulate heterogeneity.

Sayuri K. Hortsch [1,*] and Andreas Kremling [1]

[1]Specialty Division for Systems Biotechnology, Faculty of Mechanical Engineering, Technical University of Munich, Garching, Germany

[*] Corresponding author

Contents

1 Theoretical Background **2**
- 1.1 Choosing a stochastic modeling approach . . . 2
- 1.2 Chemical master equation . . . 2
- 1.3 The classical linear noise approximation . . . 3
- 1.4 Gillespie algorithm . . . 4

2 Method development **5**
- 2.1 Preliminary calculations: Means and variances of the master equation . . . 5
- 2.2 Classical rate laws and the hybrid deterministic model . . . 6
  - 2.2.1 Derivation of the hybrid deterministic model . . . 6
  - 2.2.2 Stability of the deterministic models . . . 8
- 2.3 Application of the classical and hybrid linear noise approximation to the full reaction system . . . 9
- 2.4 Application of the linear noise approximation to the reduced system . . . 10
- 2.5 Definitions of burst measures . . . 11
  - 2.5.1 Burst size . . . 11
  - 2.5.2 Burst frequency . . . 11
- 2.6 Model reduction and estimation of modes . . . 12
  - 2.6.1 Model reduction . . . 12
  - 2.6.2 Solving the CME of the reduced model . . . 13
  - 2.6.3 Estimation of modes . . . 14
- 2.7 Overview of graphical methods . . . 15

3 Quality of the novel approaches **16**
- 3.1 Comparison of Fano factors obtained from classical LNA, hybrid LNA and simulations 16
- 3.2 Multistability vs. multimodality . . . 18
- 3.3 Quality of model reduction and of the determination of modes . . . 19

4 Biological interpretations and applications 20
4.1 Extraction of burst characteristics from time course data 20
4.1.1 Regulatory systems without feedback and with linear propensities 20
4.1.2 Regulatory systems with feedback and with linear propensities 22
4.1.3 Regulatory systems with nonlinear propensities 23
4.1.4 Statistical evaluation of estimates 24
4.2 Noise in systems with saturated translation propensity 25
4.3 Analyzing the robustness of stable states in bistable systems via first passage times . . 27

5 Choice of parameter values 28

1 Theoretical Background

1.1 Choosing a stochastic modeling approach

There are different stochastic approaches for the modeling of biological circuits. The discrete chemical master equation (CME) is considered a very detailed and rather exact representation, since it describes the discrete changes in copy numbers of reaction species caused by single reactions [1]. This usually comes at the expense of analytic tractability. Exact solutions are only available for systems with limited size and/or complexity [2, 3], which is why several approximate methods exist, for example Fokker-Planck equations or Langevin equations [4, 5, 6]. Furthermore, simulation methods like the Gillespie algorithm help to gain insight into the dynamics of the model without the need for a mathematical analysis of the master equation [7]. Further ways of model simplification are often used especially in the biological context, like the elimination of certain reaction species or of intermediate reaction steps. For example, very fast reactions (e.g. the assembly of the transcription or translation machinery, protein oligomerizations) are commonly lumped into single, possibly nonlinear reaction rates, which depend on the system state (pseudo-steady-state assumption) [8]. The rates can for instance have the structure of Hill-type kinetics. Moreover, in several publications, DNA and mRNA dynamics are not considered explicitly, and protein bursts are modeled as sudden increases in copy numbers, whose magnitudes are geometrically distributed [9, 10].
In this study, we want to analyze the propagation of noise and the emergence of bursts in detail, but with a model of reasonable complexity. We will focus on translational bursts caused by the interplay of fast mRNA degradation and even more frequent translation events, which were observed experimentally in several important regulatory systems, for example in the bistable *lac* operon of *E. coli* [11, 12, 13]. Therefore, we choose to describe reactions on the mRNA and protein level using the chemical master equation, while the DNA level is neglected. We regard linear as well as nonlinear reaction propensities that are based on the pseudo-steady-state assumption. The analysis of the CME is supported by the linear noise approximation (LNA). The theoretical background of the CME, LNA, and Gillespie algorithm are explained in the following sections.

1.2 Chemical master equation

The CME is a re-formulation of the Chapman-Kolmogorov equation for Markov processes, describing the temporal change of a system's state in a probabilistic manner. Here, we regard a system with constant volume V comprising N different reaction species. The system is assumed to be homogeneously mixed, so that its state can be characterized by a vector

$\mathbf{x} = (x_1, ..., x_N)^\top$, where x_i is the current molecule copy number of the i-th reaction species. Let $\mathbf{X}(\tau)$ denote the corresponding stochastic process with time variable τ. Since the state space is discrete, the CME can be written as

$$\dot{p}_{\mathbf{x}}(\tau) = \sum_{\mathbf{x}'} \left(\pi(\mathbf{x}, \mathbf{x}')\, p_{\mathbf{x}'}(\tau) - \pi(\mathbf{x}', \mathbf{x})\, p_{\mathbf{x}}(\tau)\right), \tag{1}$$

where $p_{\mathbf{x}}(\tau)$ is the probability that $\mathbf{X}(\tau) = \mathbf{x}$, $\pi(\mathbf{x}_2, \mathbf{x}_1)$ is the transition probability per infinitesimal unit time from state $\mathbf{x}_1$ to $\mathbf{x}_2$ and the summation runs over all possible states. Put simply, the CME says that the probability of any state $\mathbf{x}$ is increased by reactions that transfer the system from other states to $\mathbf{x}$, and diminished by reactions away from $\mathbf{x}$. For a detailed derivation of the CME, see for example [1].

What remains to be defined is the explicit formulation of $\pi(.,.)$. This function only covers transitions caused by single reactions, since the infinitesimal time unit is sufficiently short so that the probability of having two or more reactions in this time interval is negligible. If R reactions are considered and the stoichiometric matrix is denoted by $\mathbf{A}$ with columns $\mathbf{a}_1, ..., \mathbf{a}_R$, then the CME can be re-written as

$$\dot{p}_{\mathbf{x}}(\tau) = \sum_{j=1}^{R} \left(w_j(\mathbf{x} - \mathbf{a}_j) \cdot p_{\mathbf{x}-\mathbf{a}_j}(\tau) \;-\; w_j(\mathbf{x}) \cdot p_{\mathbf{x}}(\tau)\right), \tag{2}$$

where $w_j(\mathbf{x})$ is the probability per infinitesimal time unit for the j-th reaction to occur (the so-called *propensity*), when the system is in state $\mathbf{x}$. The propensities are related to the classical deterministic reaction rates as follows: Let $C_i,\ i \in \{1, ..., N\}$ be the i-th reaction component and let the j-th reaction be given by

$$\sum_{i=1}^{N} \alpha_{i,j} \cdot C_i \quad \xrightarrow{k_j} \quad \sum_{i=1}^{N} \beta_{i,j} \cdot C_i, \tag{3}$$

where k_j is the (deterministic) reaction rate constant and $\alpha_{i,j}$, $\beta_{i,j}$ are the stoichiometric coefficients of the educts and products in the j-th reaction. Then $\mathbf{a}_j = (\beta_{i,j} - \alpha_{i,j})_{i=1,...,N}$ and

$$w_j(\mathbf{x}) = \frac{k_j}{V^{\sum_{i=1}^{N} \alpha_{i,j} - 1}} \prod_{i=1}^{N} \frac{x_i!}{(x_i - \alpha_{i,j})!}. \tag{4}$$

The expression $\frac{k_j}{V^{\sum_{i=1}^{N} \alpha_{i,j} - 1}}$ is the reaction rate in terms of copy numbers instead of concentrations. It scales with the volume of the system V (except for the case of first order reactions), which is plausible since the reactions occur via random encounters between molecules.

The reaction rate k_j is sometimes assumed to depend on $\mathbf{x}$. This is usually the case when a series of very fast reactions is not formulated explicitly, but lumped into one single rate.

1.3 The classical linear noise approximation

By means of the linear noise approximation (LNA), an approximate multivariate analysis of fluctuations around a deterministic stable steady state can be obtained, when a direct analysis of the probability distribution is intractable [1]. Here, we follow in large part the procedure and nomenclature proposed in [5].

In short, $\mathbf{X}(\tau)$ is replaced by $\mathbf{c}^* \cdot V + \epsilon(\tau)$, where $\mathbf{c}^*$ is a stable fixed point of the corresponding deterministic formulation

$$\dot{\mathbf{c}} = \left(\frac{1}{V} \cdot \mathbf{A} \cdot \mathbf{w}(\mathbf{c} \cdot V) \right) \Big|_{V \to \infty}, \tag{5}$$

$\mathbf{w}(\mathbf{x}) = (w_1(\mathbf{x}), ..., w_R(\mathbf{x}))^\top$ (vector of propensities), and ϵ denotes the stochastic deviation from the deterministic solution. This is inserted into the expressions $\frac{d\,\mathbb{E}[\mathbf{X}(\tau)]}{d\tau}$ and $\frac{d\,\mathbb{E}[\mathbf{X}(\tau)\,\mathbf{X}(\tau)^\top]}{d\tau}$ and the higher-order terms $O(|\epsilon|^2)$ are omitted, resulting in a linearization of reaction propensities around $\mathbf{x}^* := \mathbf{c}^* \cdot V$. The variance Σ of ϵ is then calculated for the linearized system in steady state $\left(\frac{d\,p_{\mathbf{x}}(\tau)}{d\,\tau} = 0\right)$, which serves as approximation of the local variance of $\mathbf{X}$.
Before giving the resulting mathematical formula for Σ, some further definitions need to be introduced first: Based on the stoichiometric matrix $\mathbf{A} = (a_{i,j})_{\substack{i=1,...,N,\\ j=1,...,R}}$ and the vector $\mathbf{w}(\mathbf{x})$ of the current propensities, the matrix $\mathbf{K} = (k_{i,j})_{\substack{i=1,...,N,\\ j=1,...,R}}$ is defined as

$$\mathbf{K}(\mathbf{c}) := \frac{\partial\, \mathbf{A}\, \mathbf{w}(\mathbf{c} \cdot V)}{V \cdot \partial \mathbf{c}}, \tag{6}$$

which is the Jacobian of the right hand side of the deterministic formulation (5). Furthermore, the diffusion matrix $\mathbf{D} = (d_{i,l})_{\substack{i=1,...,N,\\ l=1,...,N}}$ is given by

$$d_{i,l}(\mathbf{x}) := \sum_{j=1}^{R} a_{i,j}\, a_{l,j}\, w_j(\mathbf{x}). \tag{7}$$

If $\mathbf{D}$ is a diagonal matrix and $N = 2$, it can be shown that the covariance matrix Σ of fluctuations around the stable deterministic steady state $\mathbf{x}^*$ is approximated by

$$\begin{aligned} \Sigma = &- \frac{1}{2} \frac{d^*_{1,1}}{k^*_{1,1} + k^*_{2,2}} \left(\begin{pmatrix} 1 & 0 \\ 0 & 0 \end{pmatrix} + \frac{1}{k^*_{1,1}\, k^*_{2,2} - k^*_{1,2}\, k^*_{2,1}} \begin{pmatrix} (k^*_{2,2})^2 & -k^*_{2,1}\, k^*_{2,2} \\ -k^*_{2,1}\, k^*_{2,2} & (k^*_{2,1})^2 \end{pmatrix} \right) \\ &- \frac{1}{2} \frac{d^*_{2,2}}{k^*_{1,1} + k^*_{2,2}} \left(\begin{pmatrix} 0 & 0 \\ 0 & 1 \end{pmatrix} + \frac{1}{k^*_{1,1}\, k^*_{2,2} - k^*_{1,2}\, k^*_{2,1}} \begin{pmatrix} (k^*_{1,2})^2 & -k^*_{1,2}\, k^*_{1,1} \\ -k^*_{1,2}\, k^*_{1,1} & (k^*_{1,1})^2 \end{pmatrix} \right), \end{aligned} \tag{8}$$

where $d^*_{i,j}$ and $k^*_{i,j}$ denote the corresponding entries of $\mathbf{K}(\frac{\mathbf{x}^*}{V})$ and $\mathbf{D}(\mathbf{x}^*)$.

1.4 Gillespie algorithm

The Gillespie algorithm is a Monte-Carlo method that numerically simulates exact trajectories of the CME. It can be divided into four steps:

- Initialization:
  Initialize the vector $\mathbf{x}$ and calculate the propensities $w_j(\mathbf{x})$ of all reactions. Set $\tau = 0$.

- Monte-Carlo step:
  Randomly choose a time step $\Delta\tau$ (time until the next reaction event) and a number

$J \in \{1, ..., R\}$ (number of the reaction to occur next). Do this by drawing two independent, uniformly distributed random variables $U_1 \sim \mathcal{U}([0,1])$ and $U_2 \sim \mathcal{U}([0,1])$ and by calculating

$$\Delta\tau = \frac{1}{\sum_{j=1}^{N} w_j(\mathbf{x})} \cdot \ln\left(\frac{1}{U_1}\right) \tag{9}$$

and by choosing J which meets the condition

$$\sum_{j=1}^{J-1} w_j(\mathbf{x}) \;<\; U_2 \cdot \sum_{j=1}^{R} w_j(\mathbf{x}) \;\leq\; \sum_{j=1}^{J} w_j(\mathbf{x}) \tag{10}$$

- Update:
  Set $\tau \leftarrow \tau + \Delta\tau$ and $\mathbf{x} \leftarrow \mathbf{x} + \mathbf{a}_J$.
- Iteration:
  Go back to the Monte-Carlo step unless the stop criterion is fulfilled.

A rigorous discussion of the algorithm can for example be found in [7].

2 Method development

2.1 Preliminary calculations: Means and variances of the master equation

Let $X(\tau)$ and $Y(\tau)$ be two continuous-time stochastic processes with joint probability distribution $p_{X,Y}(\tau)$ and let $h : \mathbb{R}^2 \to \mathbb{R}$ be an arbitrary function. For the sake of simplicity, we omit (τ) in our notation. The derivative of $\mathbb{E}[h(X,Y)]$ w.r.t. τ is given by

$$\dot{\mathbb{E}}[h(X,Y)] = \sum_y \sum_x h(x,y)\, \dot{p}_{x,y}. \tag{11}$$

Based on this equation, the time derivatives of the means, variances and of the covariance can be formulated using

$$\dot{\mathrm{Var}}(X) = \dot{\mathbb{E}}[X^2] - 2\,\mathbb{E}[X]\,\dot{\mathbb{E}}[X] \tag{12}$$

and

$$\dot{\mathrm{Cov}}(X,Y) = \dot{\mathbb{E}}[X\,Y] - \mathbb{E}[X]\,\dot{\mathbb{E}}[Y] - \mathbb{E}[Y]\,\dot{\mathbb{E}}[X]. \tag{13}$$

In our case, insertion of the master equation (2) from the main text yields

$$\begin{aligned}
\dot{\mathbb{E}}[M] =& \sum_{s=0}^{\infty}\sum_{m=0}^{\infty} \Big(m\; F(s)\; p_{m-1,s} \;-\; m\; F(s)\; p_{m,s} \;+\; m\,(m+1)\; p_{m,s} \;-\; m^2\; p_{m,s} \\
& \qquad [m\; G(m)\, p_{m,s-1} \;-\; m\; G(m)\, p_{m,s} \;+\; m\,(s+1)\; p_{m,s+1} \;-\; m\; s\; p_{m,s}] \cdot \nu \Big) \\
=& \sum_{s=0}^{\infty}\sum_{m=0}^{\infty} \big(F(s) \;-\; m \big)\; p_{m,s} \\
=& \;\mathbb{E}[F(S)] - \mathbb{E}[M]
\end{aligned} \tag{14}$$

and, in an analogue manner,

$$\dot{\mathrm{Var}}(M) = \mathbb{E}[F(S)] + \mathbb{E}[M] - 2\,\mathrm{Var}(M) + 2\,\mathrm{Cov}(M, F(S)) \tag{15}$$

$$\dot{\mathbb{E}}[S] = \Big(\mathbb{E}[G(M)] - \mathbb{E}[S]\Big) \cdot \nu \tag{16}$$

$$\dot{\mathrm{Var}}(S) = \Big(\mathbb{E}[G(M)] + \mathbb{E}[S] - 2\,\mathrm{Var}(S) + 2\,\mathrm{Cov}(S, G(M))\Big) \cdot \nu \tag{17}$$

$$\dot{\mathrm{Cov}}(M,S) = \mathrm{Cov}(S, F(S)) + \nu \cdot \mathrm{Cov}(M, G(M)) - (1+\nu) \cdot \mathrm{Cov}(M,S) \tag{18}$$

For calculating steady state distributions, all time derivatives are set to 0 and the obtained system of equations is solved, which leads to the implicit relations:

$$\mathbb{E}[M] = \mathbb{E}[F(S)] \tag{19}$$

$$\mathrm{Var}(M) = \mathbb{E}[M] + \mathrm{Cov}(M, F(S)) \tag{20}$$

$$\mathbb{E}[S] = \mathbb{E}[G(M)] \tag{21}$$

$$\mathrm{Var}(S) = \mathbb{E}[S] + \mathrm{Cov}(S, G(M)) \tag{22}$$

$$\mathrm{Cov}(M,S) = \frac{\mathrm{Cov}(S, F(S)) + \nu \cdot \mathrm{Cov}(M, G(M))}{1+\nu}. \tag{23}$$

2.2 Classical rate laws and the hybrid deterministic model

2.2.1 Derivation of the hybrid deterministic model

The hybrid model is derived starting from Eqs (14) and (16), which can be written as

$$\dot{c}_m(\tau) = \frac{1}{V}\,\mathbb{E}[F(S(\tau))] - c_m(\tau) = \frac{1}{V}\sum_{r=0}^{\infty} F(r) \cdot p_r^S(\tau) - c_m(\tau) \tag{24}$$

$$\dot{c}_s(\tau) = \Big(\frac{1}{V}\,\mathbb{E}[G(M(\tau))] - c_s(\tau)\Big) \cdot \nu = \Big(\frac{1}{V}\sum_{n=0}^{\infty} G(n) \cdot p_n^M(\tau) - c_s(\tau)\Big) \cdot \nu, \tag{25}$$

where $c_m(\tau) := \frac{\mathbb{E}[M(\tau)]}{V}$ and $c_s(\tau) := \frac{\mathbb{E}[S(\tau)]}{V}$ are the expected average mRNA and protein concentrations at time τ. Furthermore, the marginal probability mass functions are denoted as $p_r^S(\tau) := \sum_{m=0}^{\infty} p_{m,r}(\tau)$ and $p_n^M(\tau) := \sum_{s=0}^{\infty} p_{n,s}(\tau)$. Their mean values are $c_s(\tau) \cdot V$ and $c_m(\tau) \cdot V$, respectively.

If F and G are linear, information on p^S and p^M is not required since the marginal distributions can be eliminated, leading to the classical rate laws that – in this case – exactly describe the dynamics of the average mRNA and protein concentrations:

$$\frac{1}{V}\sum_{r=0}^{\infty} F(r) \cdot p_r^S(\tau) = \mathcal{F}\left(\frac{1}{V}\sum_{r=0}^{\infty} r \cdot p_r^S(\tau)\right) = \mathcal{F}(c_s(\tau)), \tag{26}$$

$$\frac{1}{V}\sum_{n=0}^{\infty} G(n) \cdot p_n^M(\tau) = \mathcal{G}\left(\frac{1}{V}\sum_{n=0}^{\infty} n \cdot p_n^M(\tau)\right) = \mathcal{G}(c_m(\tau)). \tag{27}$$

However, if F and/or G are nonlinear, the equations are not valid (note that in general, $\mathbb{E}[G(M)] \neq G(\mathbb{E}[M])$ and $\mathbb{E}[F(S)] \neq F(\mathbb{E}[S])$). Moreover, the joint and marginal probability distributions might be multimodal. In order to obtain local information, we interpret multimodal distributions as superpositions of two or more unimodal ones each of which represents one cellular state. Since there is no means to determine the unimodal probability mass functions analytically, we require suitable approximations $p_r^{S,\mathrm{loc}}(\tau)$ and $p_n^{M,\mathrm{loc}}(\tau)$.

Let us first define an approximation of the local mRNA distribution $p^{M,\mathrm{loc}}(\tau)$. For the sake of simplicity, we omit the label "loc" for local (unimodal) distributions, means, and variances in the following. Z-transforming[1] the CME of the mRNA distribution in steady state

$$0 = \frac{d\,p_n^M}{d\,\tau} = \sum_{s=0}^{\infty} \big(F(s)\,p_{n-1,s} - F(s)\,p_{n,s} + (n+1)\,p_{n+1,s} - n\,p_{n,s}\big) \tag{28}$$

yields

$$\begin{aligned} 0 &= \sum_{s=0}^{\infty} \Big[F(s) \sum_{n=0}^{\infty} \frac{1-z}{z}\, z^{-n}\, p_{n,s}\Big] - z\,(z-1)\,\frac{\partial}{\partial z}\left(\sum_{n=0}^{\infty} z^{-n}\, p_n^M\right), \\ \Leftrightarrow \quad 0 &= \sum_{s=0}^{\infty} \Big[F(s) \sum_{n=0}^{\infty} z^{-n}\, p_{n,s}\Big] + z^2\,\frac{\partial}{\partial z}\left(\sum_{n=0}^{\infty} z^{-n}\, p_n^M\right) \end{aligned} \tag{29}$$

and backtransformation gives the recursion formula

$$(n+1)\,p_{n+1}^M = \sum_{s=0}^{\infty} F(s)\,p_{n,s} = \mathbb{E}[F(S)|M=n]\,p_n^M, \tag{30}$$

which can be explicitly formulated as

$$\begin{aligned} p_0^M &= \left(\sum_{n=0}^{\infty} \frac{\prod_{l=0}^{n-1} \mathbb{E}[F(S)|M=l]}{n!}\right)^{-1} \\ p_n^M &= \frac{\prod_{l=0}^{n-1} \mathbb{E}[F(S)|M=l]}{n!}\, p_0^M. \end{aligned} \tag{31}$$

These formula show that if F is almost constant in the range of local protein fluctuations, i.e. if $F \widehat{\approx} a$, then $\mathbb{E}[F(S)|M=l] \approx a \;\; \forall\, l \in \mathbb{N}_0$ and $p_n^M \approx \frac{a^n}{n!}\, e^{-a}$, or, in generalized form,

$$p_n^M \approx \frac{\mathbb{E}[M]^n}{n!}\, e^{-\mathbb{E}[M]}, \tag{32}$$

i.e. the mRNA level is approximately Poisson distributed. For nonconstant F or for systems that are not in steady state, a computationally tractable closed-form expression of $p_n^M(\tau)$ is usually not available. We therefore use the Poisson distribution (Eq (32)) as approximation in all cases. The differential equation of the protein dynamics, Eq (25), is hence replaced by:

$$\dot{c}_s = \Big(\frac{1}{V} \sum_{n=0}^{\infty} G(n) \cdot \frac{(c_m\,V)^n}{n!} e^{-c_m\,V} - c_s\Big) \cdot \nu = (\bar{\mathcal{G}}(c_m) - c_s) \cdot \nu. \tag{33}$$

Obviously, this approach leads to a local averaging of the function G, cf. Figure A.

[1] Z-transform of a function $h : \mathbb{N}_0 \to \mathbb{R},\; n \mapsto h(n)$ has the form $\hat{h}(z) = \mathcal{Z}(h)(z) = \sum_{n=0}^{\infty} z^{-n}\, h(n)$.

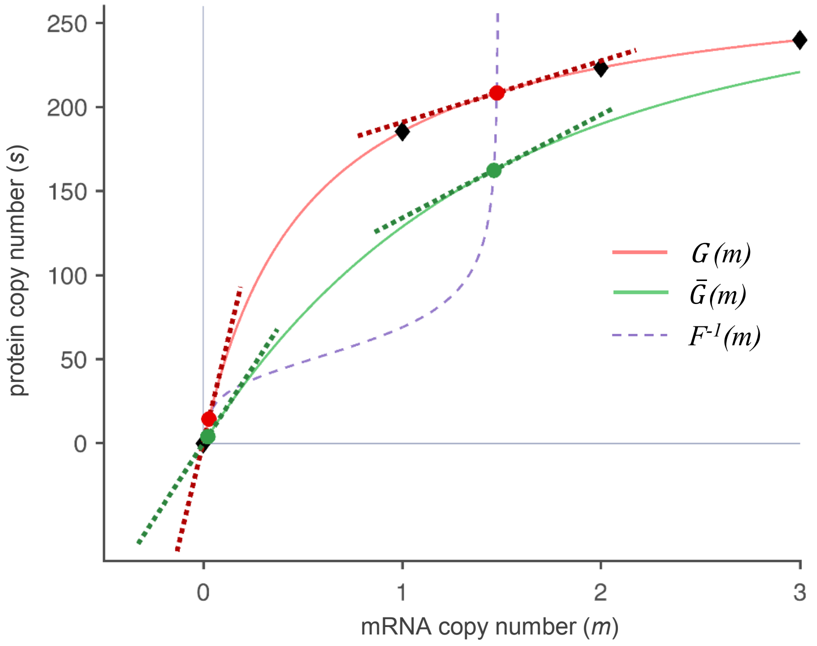

Figure A: **Continuous translation rate according to the classical and hybrid deterministic model.** While the translation rate G of the classical model (red line) is an interpolation of the discrete function values (black diamonds), the translation rate $\bar{G}$ of the hybrid deterministic model (green line) is a local average (weighted sum according to a Poisson distributed mRNA copy number with mean m). The exemplary fixed points (intersection points with F^{-1}, dashed purple line) of the classical (red filled circles) and hybrid (green filled circles) models diverge significantly. In addition, the local derivatives of G and $\bar{G}$ (dashed colored lines) at the respective fixed points are different, which has a great impact on the calculation of the Fano factors using the LNA. Functions and parameters are the same as in Fig 2 of the main text.

It would now make sense to choose a unimodal approximation of $p^{S,\mathrm{loc}}$ as well, leading to a local averaging of the function F around $\mathbb{E}[S]$. However, if the protein copy number is assumed to be locally Poisson distributed, $\mathbb{E}[F(S)]$ might be approximately equal to $F(\mathbb{E}[S])$, since the average protein level is usually high. As the variance of the real local distribution may vary considerably depending on the circuit and as it cannot be easily assessed *a priori*, we just replace Eq (24) by the classical macroscopic reaction rate formulation

$$\dot{c}_m = \mathcal{F}(c_s) - c_m. \tag{34}$$

This also leads to reasonable compuational complexity of the deterministic model.

2.2.2 Stability of the deterministic models

Let (c_m^*, c_s^*) be a fixed point of the (classical or hybrid) deterministic model. The Jacobian J of the right hand-side of the ODE system, evaluated at (c_m^*, c_s^*), reads

$$J = \begin{pmatrix} -1 & f \\ g \cdot \nu & -\nu \end{pmatrix} \tag{35}$$

with $f := \frac{d\,\mathcal{F}(c_s)}{d\,c_s}|_{c_s=c_s^*}$, $g := \frac{d\,\mathcal{G}(c_m)}{d\,c_m}|_{c_m=c_m^*}$ in case of the classical deterministic model and $g := \frac{d\,\bar{\mathcal{G}}(c_m)}{d\,c_m}|_{c_m=c_m^*}$ in case of the hybrid deterministic model.

For (c_m^*, c_s^*) to be stable, the trace of J needs to be negative and the determinant needs to be positive. The first condition is always fulfilled, while the second condition holds if $f \cdot g < 1$. In this case, the expression $(\text{trace}(J))^2 - 4 \cdot \det(J)$ is always positive, so that (c_m^*, c_s^*) turns out to be a stable node.

2.3 Application of the classical and hybrid linear noise approximation to the full reaction system

In the following, the linear noise approximation is applied to the full reaction system. The following formula are valid for both the classical and the hybrid LNA; the only difference lies in the values of the deterministic stable steady states m^* and s^* and in those of the local derivatives f and g.
The vectors and matrices defined in Section 1.3 read:

$$\mathbf{A} = \begin{pmatrix} 1 & -1 & 0 & 0 \\ 0 & 0 & 1 & -1 \end{pmatrix} \tag{36}$$

$$\mathbf{w}(m^*, s^*) = \begin{pmatrix} m^* & m^* & \nu \cdot s^* & \nu \cdot s^* \end{pmatrix}^\top \tag{37}$$

$$\mathbf{K}(c_m^*, c_s^*) = \begin{pmatrix} -1 & f \\ \nu \cdot g & -\nu \end{pmatrix} \tag{38}$$

$$\mathbf{D}(m^*, s^*) = \begin{pmatrix} 2\, m^* & 0 \\ 0 & 2\, \nu \cdot s^* \end{pmatrix} \tag{39}$$

The approximated variance-covariance matrix Σ of (M, S) has the form

$$\begin{aligned} \Sigma &= m^* \frac{1}{1+\nu} \left(\begin{pmatrix} 1 & 0 \\ 0 & 0 \end{pmatrix} + \frac{\nu}{1 - f\, g} \begin{pmatrix} 1 & g \\ g & g^2 \end{pmatrix} \right) \\ &+ s^* \frac{\nu}{1+\nu} \left(\begin{pmatrix} 0 & 0 \\ 0 & 1 \end{pmatrix} + \frac{1}{\nu(1 - f\, g)} \begin{pmatrix} f^2 & f \\ f & 1 \end{pmatrix} \right). \end{aligned} \tag{40}$$

A reformulation of $\Sigma_{1,1}$ and $\Sigma_{2,2}$ yields the following approximate expressions for $\text{Var}(M)$ and $\text{Var}(S)$:

$$\text{Var}(M) \approx m^* \left[1 + \frac{f}{g^{-1} - f} \left(\frac{\nu}{1+\nu} + \frac{1}{1+\nu} \cdot r\, \frac{f}{g} \right) \right] \tag{41}$$

$$\text{Var}(S) \approx s^* \left[1 + \frac{1}{g^{-1} - f} \left(\frac{f}{1+\nu} + \frac{\nu}{1+\nu} \cdot \frac{g}{r} \right) \right] \tag{42}$$

in a domain around (m^*, s^*), where the right hand sides correspond to the variances of the linearized system.

2.4 Application of the linear noise approximation to the reduced system

The CME of the reduced reaction system (Eq (13) in the main text) is given by

$$\begin{aligned}\frac{d\,p_s}{d\tau} &= \sum_{b=0}^{s} F(s-b)\ \mathrm{Geo}_{\alpha(F(s-b))}(b)\ p_{s-b} - F(s)\,p_s \\ &+\ \big[(s+1)\,p_{s+1} -\ s\,p_s\,\big]\cdot\nu \end{aligned} \tag{43}$$

and its mean obeys (with $F(s)=0$ if $s<0$):

$$\begin{aligned}\frac{d\,\mathbb{E}[S]}{d\tau} &= \sum_{s=0}^{\infty}\sum_{b=0}^{\infty} s\,F(s-b)\ \mathrm{Geo}_{\alpha(F(s-b))}(b)\ p_{s-b} - s\,F(s)\,p_s + \big(s\,(s+1)\,p_{s+1} -\ s^2\,p_s\,\big)\cdot\nu \\ &= \sum_{s=0}^{\infty}\sum_{b=0}^{\infty} b\,F(s)\,\mathrm{Geo}_{\alpha(F(s))}(b)\ p_s \ -\ \sum_{s=0}^{\infty} s\,p_s\cdot\nu\ =\ \sum_{s=0}^{\infty} F(s)\,\alpha(F(s))\ p_s\ -\ \mathbb{E}[S]\cdot\nu \\ &= \Big(\sum_{s=0}^{\infty}\bar{G}(F(s))\ p_s\ -\ \mathbb{E}[S]\Big)\cdot\nu\ =\ \big(\mathbb{E}[\bar{G}(F(s))]\ -\ \mathbb{E}[S]\big)\cdot\nu. \end{aligned} \tag{44}$$

Again, we consider *local* mean values and approximate:

$$\sum_{s=0}^{\infty}\bar{G}(F(s))\,p_s\ =\ \sum_{n=0}^{\infty}G(n)\sum_{s=0}^{\infty}\frac{F(s)^n}{n!}\,e^{-F(s)}\,p_s \approx \sum_{n=0}^{\infty}G(n)\frac{F(\mathbb{E}[S])^n}{n!}\,e^{-F(\mathbb{E}[S])}\ =\ \bar{G}(F(\mathbb{E}[S])),$$

(which holds exactly if F is constant), so that the deterministic formulation

$$\frac{dc_s}{d\tau} = \big(\bar{\mathcal{G}}(F(c_s)) - c_s\big)\cdot\nu \tag{45}$$

is obtained. Its fixed points c_s^* correspond to those of the full hybrid deterministic model. Linearization is thus performed around the same values.

Next, the vectors and matrices that are required for LNA are collected. Since bursts of any size can occur with a certain probability, the stochastic matrix $\mathbf{A}$ has infinite dimensions.

$$\mathbf{A}\ =\ \begin{pmatrix}-1 & 0 & 1 & 2 & 3 & \dots\end{pmatrix} \tag{46}$$

$$\mathbf{w}(s^*)\ =\ \begin{pmatrix}\nu\cdot s^* & \mathrm{Geo}_{r\nu}(0)\cdot F(s^*) & \mathrm{Geo}_{r\nu}(1)\cdot F(s^*) & \mathrm{Geo}_{r\nu}(2)\cdot F(s^*) & \dots\end{pmatrix}^{\top} \tag{47}$$

$$\mathbf{K}(c_s^*)\ =\ g\cdot f\cdot\nu\ -\ \nu \tag{48}$$

$$\begin{aligned}\mathbf{D}(s^*)\ &=\ \nu\cdot s^*\ +\ \sum_{b=0}^{\infty} b^2\,\mathrm{Geo}_{r\nu}(b)\cdot F(s^*) \\ &=\ \nu\cdot s^*\ +\ r\,\nu\,(1\ +\ 2\,r\,\nu)\cdot F(s^*) \\ &=\ 2\cdot s^*\,\nu\cdot(1\ +\ r\,\nu) \end{aligned} \tag{49}$$

Here, $g := \frac{d\bar{\mathcal{G}}(c_m)}{d\,c_m}\big|_{c_m=\mathcal{F}(c_s^*)}$ and $r := \frac{s^*}{F(s^*)}$. With $\Sigma\ =\ -\frac{D^*}{2\,K^*}$, the approximate local variance reads

$$\mathrm{Var}(S)\ = s^*\cdot\frac{1\ +\ r\,\nu}{1\ -\ f\,g}\ =\ s^*\left[1+\frac{1}{g^{-1}-f}\left(f+\frac{r\,\nu}{g}\right)\right]. \tag{50}$$

2.5 Definitions of burst measures

2.5.1 Burst size

The burst size $\alpha(m)$ is defined as the mean number of proteins produced during a burst, when the average level around which the mRNA copy number fluctuates immediately before a burst is equal to m. Again, we assume that mRNA fluctuations follow a Poisson distribution, cf. Eq (32). As shown before, this holds exactly in a system with a locally constant transcription propensity F, which is in steady state. The burst is triggered by the formation of an additional mRNA molecule and ends with its degradation. During that time, the distribution of the remaining mRNA molecules is assumed to be unaffected. Hence, the average propensity with which proteins are translated from the mRNA molecule that causes the burst is equal to $\sum_{n=0}^{\infty} \frac{G(n+1)}{n+1} \nu \frac{m^n}{n!} e^{-m} = \frac{\bar{G}(m)}{m} \nu$.
When b denotes the number of proteins translated from that specific mRNA molecule (burst proteins), the CME of the reaction

$$\emptyset \xrightarrow{\frac{\bar{G}(m)}{m}\nu} \text{Burst proteins} \tag{51}$$

is given by:

$$\begin{aligned} \dot{p}_b &= \frac{\bar{G}(m)}{m} \nu \, p_{b-1} - \frac{\bar{G}(m)}{m} \nu \, p_b, \\ p_b(0) &= \delta_b(\{0\}). \end{aligned} \tag{52}$$

$\delta_{.}(\{.\})$ denotes the Dirac measure. Let B be the stochastic variable of b. During the burst, its mean value follows the equation

$$\mathbb{E}[B(\tau)] = \frac{\bar{G}(m)}{m} \nu \, \tau. \tag{53}$$

The lifetime τ_m of a mRNA molecule is exponentially distributed with parameter 1 (inverse of the mRNA degradation rate in the scaled system). Hence, the burst size is given by

$$\alpha(m) := \mathbb{E}\left[\mathbb{E}[B(\tau_m)]\right] = \int_0^{\infty} \frac{\bar{G}(m)}{m} \nu \, \tau_m \, e^{-\tau_m} \, d\tau_m = \frac{\bar{G}(m)}{m} \nu. \tag{54}$$

2.5.2 Burst frequency

The burst frequency $\omega(s)$ is the mean number of bursts (i.e., of transcribed mRNA molecules) during the lifetime of a protein, where s is the mean copy number of proteins during that interval. Protein lifetime is exponentially distributed with mean $1/\nu$. Like in the formulation of the hybrid deterministic model, the average transcriptional propensity is approximated by the point evaluation $F(s)$. The burst frequency can now be determined by a calculation analogous to the burst size:

$$\omega(s) = \int_0^{\infty} F(s) \, \tau_s \, \nu \, e^{-\tau_s \nu} \, d\tau_s = \frac{F(s)}{\nu}. \tag{55}$$

2.6 Model reduction and estimation of modes

2.6.1 Model reduction

Let $\nu \ll 1$, so that mRNA molecules are degraded very quickly compared to the protein. In this case, all translation events belonging to one burst occur in a temporally highly condensed manner (if the reference time-scale is determined by protein degradation). Our goal is a model reduction that solely regards the protein level:

$$\begin{array}{lrcll} \text{reaction 1:} & \emptyset & \xrightarrow{F(s)\cdot P(b)} & b \cdot \text{Protein}, & b = 0, 1, 2, ... \\ \text{reaction 2:} & \text{Protein} & \xrightarrow{\nu} & \emptyset & \end{array} \tag{56}$$

Here, $P(b)$ is the probability of a burst of size b, which needs to be specified in the following:

If G is linear, the burst size α does not depend on the current mRNA level. We recall the reaction in which the burst proteins are generated:

$$\emptyset \xrightarrow{\alpha} \text{Burst proteins}, \tag{57}$$

cf. Eq (51). The burst ends with the degradation of the responsible mRNA molecule (propensity equal to 1). A burst of size b is hence generated when b protein formation events occur before the mRNA molecule is degraded. As a consequence,

$$P(b) = \left(\frac{\alpha}{1+\alpha}\right)^b \frac{1}{1+\alpha} =: \text{Geo}_\alpha(b), \tag{58}$$

i.e. the burst size is geometrically distributed for linear G.

If G is nonlinear, the current average mRNA copy number determines the burst size $\alpha(m)$. In order to eliminate the mRNA level, assumptions about m need to be made. As an approximation, we assume that between bursts, $m = F(s)$, where s is the current protein copy number. This assumption is valid either if the overall mRNA dynamics are so fast that the mRNA distribution adapts quickly to the current protein copy number (pseudo steady state), or if F is constant in the range of protein fluctuations (different expression states are again considered separately), so that the mRNA distribution is independent of the protein level. Furthermore, we assume that the mRNA distribution does not change significantly during a burst. Then, the translation of burst proteins occurs with propensity $\alpha(F(s))$, and the burst size is again geometrically distributed:

$$P(b) = \left(\frac{\alpha(F(s))}{1+\alpha(F(s))}\right)^b \frac{1}{1+\alpha(F(s))} = \text{Geo}_{\alpha(F(s))}(b). \tag{59}$$

Now, we can formulate the CME of the reduced model:

$$\begin{aligned} \frac{dp_s(\tau)}{d\tau} &= \sum_{b=0}^{s} F(s-b) \cdot \text{Geo}_{\alpha(F(s-b))}(b) \cdot p_{s-b} \, - \, F(s)\, p_s \\ &\quad + \, (s+1)\, \nu\, p_{s+1} \, - \, s\, \nu\, p_s. \end{aligned} \tag{60}$$

2.6.2 Solving the CME of the reduced model

Let us first assume that the mean burst size $\alpha(F(s))$ is constant with value $\alpha \in \mathbb{R}_{\geq 0}$, which is the case in systems without feedback (F constant) or for linear translation rates G. The steady-state condition of the CME can be formulated as

$$\sum_{b=0}^{\infty} F(s-b) \cdot \mathrm{Geo}_{\alpha}(b) \cdot p_{s-b} \;-\; F(s)\, p_s \;=\; -\nu\left((s+1)\, p_{s+1} \;-\; s\, p_s\right)$$
$$\Leftrightarrow \quad \left(\left(\mathrm{Geo}_{\alpha} - \delta_{\cdot}(\{0\})\right) \,*\, F\, p\right)(s) \;=\; -\nu\left((s+1)\, p_{s+1} \;-\; s\, p_s\right), \tag{61}$$

where $F(s) = 0 \;\forall\; s < 0$, $\delta_{\cdot}(.)$ is again the Dirac measure, and $*$ denotes a discrete convolution.

The steady state distribution of the reduced model can now be calculated using Z-transform (cf. Section 2.2.1). By applying some general rules, we obtain for the LHS of the equation:

$$\mathcal{Z}\left(\left(\mathrm{Geo}_{\alpha} - \delta_{\cdot}(\{0\})\right) \,*\, F\, p\right)(z) \;=\; \left(\mathcal{Z}(\mathrm{Geo}_{\alpha})(z) - \mathcal{Z}(\delta_{\cdot}(\{0\}))(z)\right) \cdot \mathcal{Z}(\,F\, p)(z) \;=$$
$$= \left(\mathcal{Z}(\mathrm{Geo}_{\alpha})(z) - 1\right) \cdot \left(\hat{F} * \hat{p}\right)(z) \;=\; \left(\sum_{s=0}^{\infty} \frac{1}{1+\alpha}\left(\frac{\alpha}{1+\alpha}\right)^{s} z^{-s} \;-\; 1\right) \cdot \left(\hat{F} * \hat{p}\right)(z) \;=$$
$$= \left(\frac{1}{1+\alpha}\sum_{s=0}^{\infty}\left(\frac{\alpha}{z(1+\alpha)}\right)^{s} \;-\; 1\right) \cdot \left(\hat{F} * \hat{p}\right)(z) \;=\; \left(\frac{1}{1+\alpha} \cdot \frac{1}{1-\frac{\alpha}{z\,(1+\alpha)}} \;-\; 1\right) \cdot \left(\hat{F} * \hat{p}\right)(z) \;=$$
$$= -\;\frac{\alpha\,(z-1)}{\alpha\,(z-1)+z} \cdot \left(\hat{F} * \hat{p}\right)(z). \tag{62}$$

Moreover, Z-transform of the RHS (divided by $-\nu$) gives

$$\sum_{s=0}^{\infty} z^{-s}\,\left((s+1)\, p_{s+1} \;-\; s\, p_s\right) \;=\; z\,(z\;-\;1)\,\frac{1}{z}\sum_{s=0}^{\infty} z^{-s}\, s\, p_s \;=\; -z\,(z\;-\;1)\,\partial_z \hat{p}(z). \tag{63}$$

All in all, the Z-transform of the whole steady state equation reads

$$z\,(z\;-\;1)\,\partial_z \hat{p}(z) \;=\; -\frac{\alpha\,(z-1)}{\alpha\,(z-1)+z} \cdot \frac{1}{\nu}\left(\hat{F} * \hat{p}\right)(z) \tag{64}$$

or, after some simplification:

$$-\,z\,(z\;-\;1)\,\partial_z \hat{p}(z) \;-\; \frac{z^2}{\alpha}\,\partial_z \hat{p}(z) \;=\; \frac{1}{\nu}\left(\hat{F} * \hat{p}\right)(z). \tag{65}$$

It is straightforward to show that $-\frac{z^2}{\alpha}\,\partial_z \hat{p}(z) \;=\; \frac{1}{\alpha}\,\mathcal{Z}((s\;+\;1)\, p_{s+1})$. Therefore, backtransformation yields

$$(s+1)\, p_{s+1} \;-\; s\, p_s \;+\; \frac{1}{\alpha}\,(s\;+\;1)\, p_{s+1} \;=\; \frac{F(s)}{\nu}\, p_s, \tag{66}$$

from which follows the simplified recursive steady state condition

$$(s+1)\, p_{s+1} \;=\; \frac{\alpha}{1\;+\;\alpha}\left(\frac{F(s)}{s\,\nu} \;+\; 1\right) s\, p_s, \quad s > 0,$$
$$p_1 \;=\; \frac{\alpha}{1\;+\;\alpha}\,\frac{F(0)}{\nu}\, p_0. \tag{67}$$

Now, the explicit steady-state-distribution can be retrieved:

$$p_s = \frac{F(0)}{s\,\nu}\,p_0 \left(\frac{\alpha}{1+\alpha}\right)^s \prod_{i=1}^{s-1}\left(\frac{F(i)}{i\,\nu} + 1\right). \tag{68}$$

For bursts that depend on the protein level, this explicit calculation is not possible. We assume that in this case, the approximate relations

$$\begin{aligned}(s+1)\,p_{s+1} &= \frac{\alpha(F(s))}{1 + \alpha(F(s))}\left(\frac{F(s)}{s\,\nu} + 1\right) s\,p_s, \quad s > 0\\ p_1 &= \frac{\alpha(F(0))}{1 + \alpha(F(0))}\,\frac{F(0)}{\nu}\,p_0\end{aligned} \tag{69}$$

and

$$p_s = \frac{F(0)}{s\,\nu}\,p_0\,\frac{\alpha(F(0))}{1+\alpha(F(0))}\prod_{i=1}^{s-1}\left[\frac{\alpha(F(i))}{1+\alpha(F(i))}\cdot\left(\frac{F(i)}{i\,\nu} + 1\right)\right]. \tag{70}$$

hold, that have been motivated from Eqs (67) and (68). By inserting these approximate expressions into the CME of the reduced system (60), it can be shown that they approximately fulfill the steady state condition if $\alpha(F(s)) \approx \alpha(F(s+1))\ \forall s$.

2.6.3 Estimation of modes

A local maximum (mode) of the probability distribution occurs at a positive protein copy number $s > 0$ if $p_{s+1} \leq p_s$ and $p_{s-1} \leq p_s$. Using Eq (69), we conclude that s then fulfills the condition

$$s = \lceil\sigma\rceil \qquad \text{where} \quad \sigma \in \mathbb{R}, \quad \sigma + \alpha(F(\sigma)) + 1 = \frac{\alpha(F(\sigma))\,F(\sigma)}{\nu}. \tag{71}$$

Here, $\lceil.\rceil$ is the ceiling function.
A further mode is located at $s = 0$ if $p_1 < p_0$, i.e. if

$$\alpha(F(0)) + 1 > \frac{\alpha(F(0))\,F(0)}{\nu}. \tag{72}$$

2.7 Overview of graphical methods

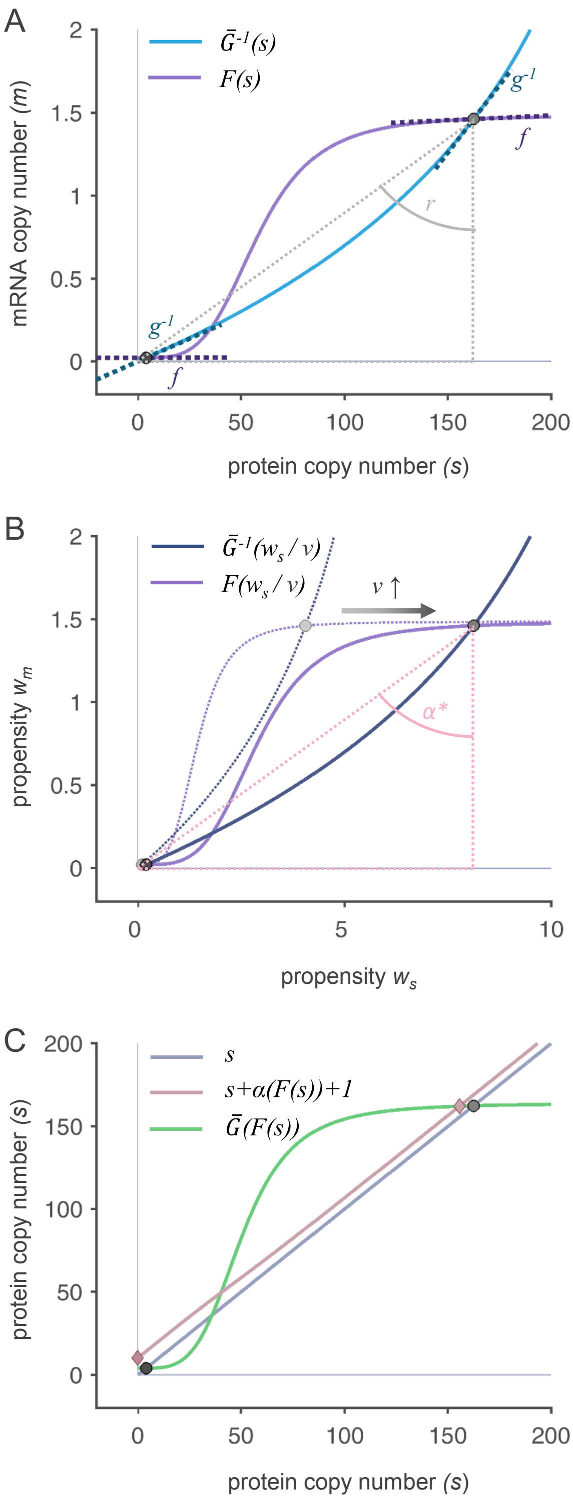

Figure B: **Graphical evaluation methods.** (A): Deterministic phase plot. (B): Propensity plot. (C): Plot for the determination of modes.

In Figure B, three graphical methods are shown with which noise can be characterized. In the protein-mRNA phase plot (A), the mRNA nullcline (purple line) and the protein nullcline (blue line) are plotted. The outer intersection points (grey circles) mark the stable deterministic steady states. From the plot, one can read the local derivatives f and g (dashed lines) as well as the protein-to-mRNA ratio r at each stable fixed point. This way, one can qualitatively predict how a modulation of the shapes of F and G influences the local Fano factors. Obviously, choosing a nonlinear G allows more flexible adjustment of noise (otherwise, g and r would be identical and constant). Note that ν does not have an influence on the graphs in the first plot, in contrast to the propensity plot shown in (B). It is similar to the phase plot, but with different scaling. The local average burst size α^* is equal to the w_s-to-w_m-ratio at the outer intersection points. A variation of ν stretches or compresses the plotted functions equally. This has only an effect on w_s, while the mRNA dynamics stay the same. Since $w_s = \bar{G}(w_m) \cdot \nu$, the effect of ν on w_s is linear. Again, nonlinearity of G is a necessary condition for generating different burst sizes in the two expression states.

The third plot is applicable if $\nu \ll 1$. Here, the locations of the local mean values (grey circles) can be compared with the locations of the modes (pink diamonds). The larger the local burst size $\alpha(F(s))$ is, the larger are the deviations between modes and the corresponding local mean values.

Here, the functions and parameters from Fig 2 of the main text have been used. In (B), a shift from $\nu = 0.025$ to $\nu = 0.05$ is illustrated. In (C), $\nu = 0.05$ was chosen so that the shift between local means and modes becomes better visible. For better readability, one may vary the scaling of the axes (e.g. log-log-scale). However, the plots shown here allow a more intuitive understanding of the connection between circuit properties and noise.

3 Quality of the novel approaches

3.1 Comparison of Fano factors obtained from classical LNA, hybrid LNA and simulations

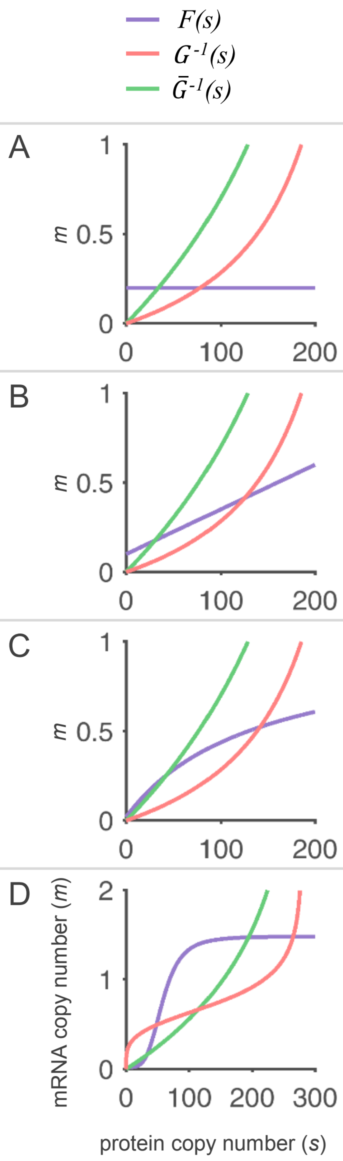

	s^*, v = 0.01	s^*, v = 0.1	$\eta^*(S)$, v = 0.01	$\eta^*(S)$, v = 0.1
A (simulation)	34.3 ± 0.4	34.2 ± 1.2	2.5 ± 0.1	14.6 ± 0.7
A (classical LNA)	78.7		3.0	19.5
A (hybrid LNA)	34.3		2.4	14.3
B (simulation)	30.4 ± 0.6	29.1 ± 1.5	4.2 ± 0.2	23.2 ± 1.5
B (classical LNA)	125.1		3.3	16.3
B (hybrid LNA)	30.5		4.2	24.5
C (simulation)	37.9 ± 1.3	20.8 ± 2.8	7.7 ± 0.6	42.0 ± 3.0
C (classical LNA)	141.7		2.2	9.2
C (hybrid LNA)	42.6		6.5	37.1
D (simulation)	194.2 ± 1.4<br>5.0 ± 0.3	193.9 ± 1.7<br>3.3 ± 2.0	1.5 ± 0.1<br>3.4 ± 0.4	5.7 ± 0.4<br>11.5 ± 6.3
D (classical LNA)	265.3<br>0.0		1.0<br>4.2	1.0<br>30.1
D (hybrid LNA)	194.2<br>4.8		1.4<br>3.2	4.5<br>20.6

Figure C: **Comparing the classical and hybrid LNA.** The s-m-plots on the left visualize the difference in the fixed points of the classical and hybrid deterministic model. In the table on the right, the estimated fixed points and protein Fano factors of both approaches (red: classical LNA, green: hybrid LNA) are compared to values extracted from simulations (blue; estimates ± SEMs of 10^2 repeats are shown). Different combination of functions have been tested. (A): Constant F, saturated G. (B): Linear, increasing F, saturated G. (C): Saturated F and G. (D): Sigmoid F and G. Here, the estimates for both steady states are given (upper values: active state; lower values: inactive state). Functions and parameters are listed in the text.

In addition to the comparison done in Fig 2 of the main text, we have evaluated further combinations of F and G in order to test the quality of the LNA approaches. In Figure C, the phase plots of the systems are shown. The functions and parameters are as follows:

A.	constant F, concave G:	$F(s) = 0.2,$	$G(m) = 281\,\frac{m}{m+0.51}$
B.	linear F, concave G:	$F(s) = 0.1 + 0.0025 \cdot s,$	$G(m) = 281\,\frac{m}{m+0.51}$
C.	concave F and G:	$F(s) = 0.02 + \frac{s}{s+140},$	$G(m) = 281\,\frac{m}{m+0.51}$
D.	sigmoid F and G:	$F(s) = 0.022 + 1.46\,\frac{s^4}{s^4+58^4},$	$G(m) = 281\,\frac{m^4}{m^4+0.73^4}$

The parameter ν has been varied ($\nu = 0.01$ and $\nu = 0.1$). This does not affect the phase plots, so that the fixed points of the deterministic models remain the same. The tables on the right list the estimates of s^* and $\eta^*(S)$ obtained with the classical and hybrid LNA. Moreover, the corresponding values that have been extracted from simulations are shown. In order to generate these values, protein time courses have been simulated with final reaction time $t_{\max} = 10^5$ for $\nu = 0.01$ and $t_{\max} = 10^4$ for $\nu = 0.1$. Then, their means and variances have been evaluated for systems (A) to (C). In case of the bistable system (D), phases have been identified visually in every time course in which the system was clearly in one of the two expression states, so that the states could be evaluated separately. For $\nu = 0.1$, the system switches quickly to the high expression state, so that the estimates for the low expression states are of limited significance and have large standard errors.
System (A) serves as a control for the hybrid deterministic model, since F is constant and therefore, the model is expected to exactly predict the location of the mean value. This is indeed the case, while the steady state of the classical approach deviates significantly. Despite the nonlinearity of G, the hLNA also predicts the Fano factor very well.
In System (B), F is given by a linear, positively increasing function. The quality of the hybrid approach is expected to be reduced as the mRNA distribution is not exactly Poisson anymore. However, the deviation is moderate (data not shown) and thus the quality of the hybrid LNA is still very good. Again, all estimates obtained from the classical LNA are clearly worse.
In (C), F describes a saturation curve. Interestingly, the simulated local mean values are different for $\nu = 0.01$ and for $\nu = 0.1$. This can be explained as follows: In a way similar to the averaging of the translation rate G in the range of mRNA fluctuations that has been performed in the hybrid deterministic approach, an averaging of F could be done in the range of protein fluctuations. For larger ν, protein fluctuations increase, so that the averaging effect is expected to be stronger. By smoothing the graph of F (purple line) in the phase plot to various degrees, one can imagine that the fixed point of the hybrid deterministic model would move towards the simulated ones. Therefore, an extension of our hybrid approach that calculates an effective transcription rate $\bar{F}$ is expected to yield even better predictions. However, as we have mentioned before, the range of protein fluctuations cannot be estimated *a priori* (a Poisson distribution is usually too narrow and does not lead to significant differences in F and $\bar{F}$). Nevertheless, with our hybrid approach, all estimates are in a realistic range and clearly outperform the classical approach.
In (D), a hypothetical, bistable system with sigmoid F and G is shown. Note that $\bar{G}$ is not a sigmoid function anymore. F is almost constant in a domain around the fixed points. Again, the hybrid approach yields better noise estimates for both expression states, compared to the classical one.

3.2 Multistability vs. multimodality

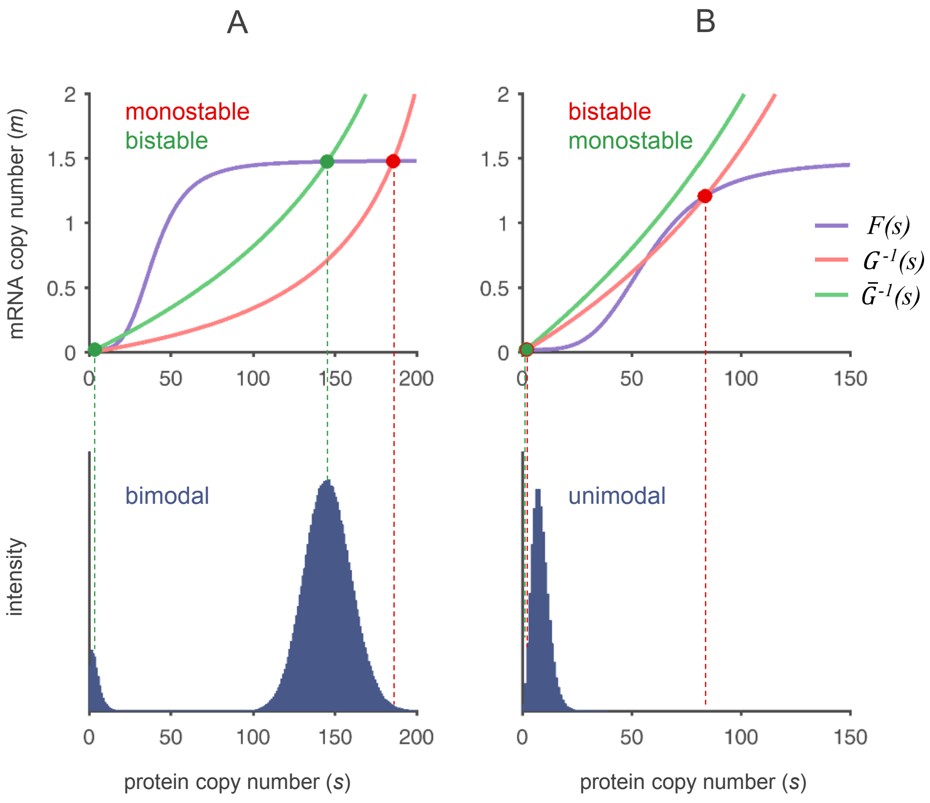

Figure D: **Differences in qualitative stability properties of classical and hybrid deterministic models.** In (A) and (B), two different systems are considered with sigmoid F and saturated G. The upper part shows the phase plots of the deterministic models. Red filled circles mark the locations of the fixed points according to the classical rate laws, while green filled circles mark those of the hybrid deterministic model. Due to the difference in G and $\bar{G}$, the number of steady states is different. The lower plots show histograms of the protein distribution obtained from simulations. (A): The system is bimodal. According to the hybrid model, it is bistable, while the classical model predicts monostability. $F(s) = 0.022 + 1.46\frac{s^4}{s^4+45^4}$, $G(m) = 250\frac{m}{m+0.51}$. (B): The protein distribution is unimodal, in accordance with the monostability of the hybrid model. However, the classical approach predicts bistability. $F(s) = 0.1 + 1.46\frac{s^4}{s^4+58^4}$, $G(m) = 281\frac{m}{m+2.57}$.

Due to the possibly large difference between G and $\bar{G}$, it is fair to assume that the classical and the hybrid deterministic approaches might predict distinct stability behaviors for the same system. Two examples are shown in Figure D, where one of the approaches is monostable, while the other one is bistable. Interestingly, the number of fixed points in the hybrid model coincides with the number of modes in the simulated protein distribution, and their locations are similar as well. Here, the distribution has been obtained from time courses of multiple simulations that have been initialized at different mRNA and protein copy numbers and stopped at different times in order to capture all stable expression states.
However, in Fig 4B of the main text, a system has been shown that is monostable according to the hybrid deterministic model, but whose distribution is bimodal. In this case, bimodality emerges due to large protein bursts that cause a significant right-skewness of the distribution.

The distribution accumulates at low protein copy numbers, forming a second peak. This peak is not connected with any deterministic stable state. Changes in multimodality through large bursts have been discussed more extensively in [14]. Apart from this phenomenon that cannot be captured by deterministic models, we expect that the hybrid model is able to predict multimodality more reliably than the classical model.

3.3 Quality of model reduction and of the determination of modes

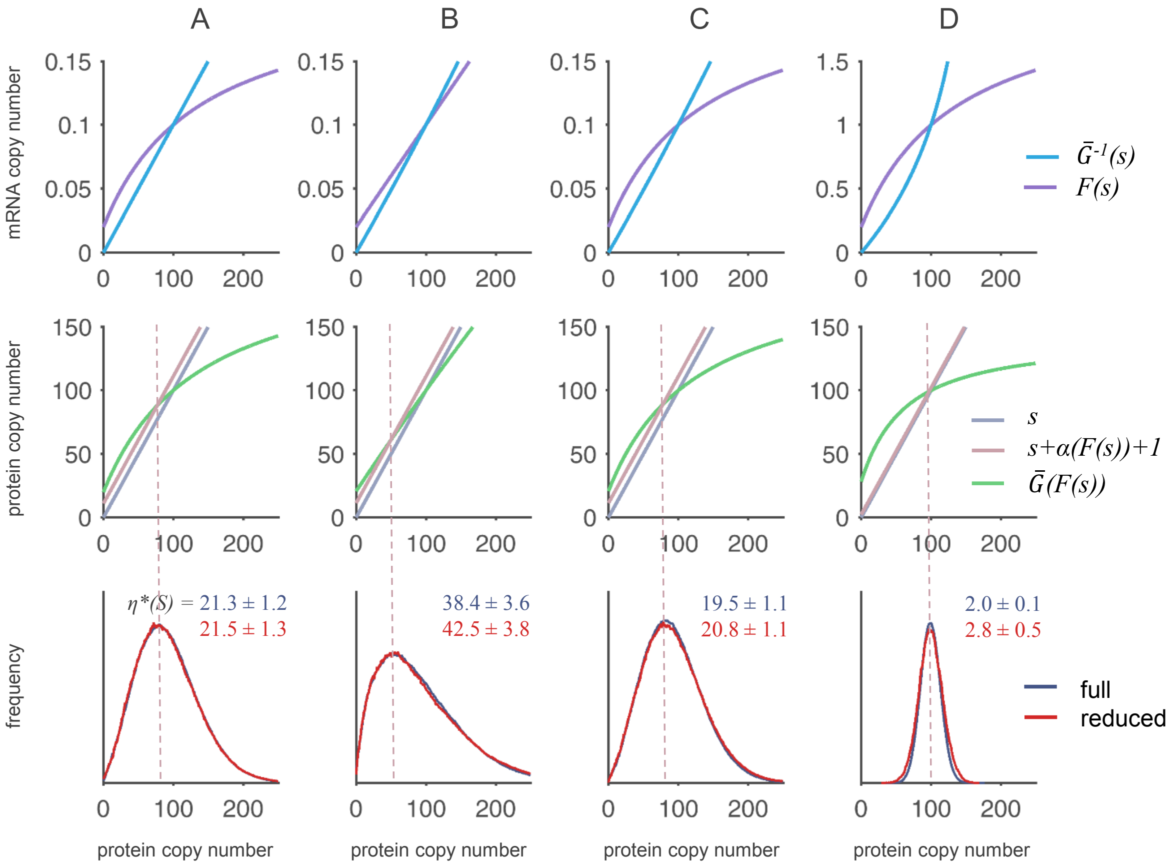

Figure E: **Quality of model reduction and mode prediction.** Four different reaction systems that are specified in the text are evaluated. Top: s-m-phase-plots. Middle part: Plots for the prediction of modes. Bottom: Histograms of the full and reduced models. To generate the distributions, protein time courses of each (full or reduced) system have been simulated with final reaction time $t_{\max} = 10^5$. Protein Fano factors of the time courses with their SEMs are given (obtained from at least 30 repeats). Histograms of full and reduced systems are nearly congruent if $\bar{G}$ is linear as in system (A), while the distribution of the reduced system is broader if $\bar{G}$ is strongly concave as in (D). Nonlinearity of F does not hamper the quality of model reduction (cf. (B) and (C)). The prediction of modes worked well in all cases.

In order to further assess the quality of our model reduction technique, simulations of full and reduced reaction systems have been performed. Here, $\nu = 0.01 \ll 1$. The following circuits have been tested:

A.	concave F, linear G:	$F(s) = 0.02 + 0.192\,\frac{s}{s+140}$,	$G(m) = 1000 \cdot m$
B.	linear F, concave G:	$F(s) = 0.02 + 0.0008 \cdot s$,	$G(m) = 1153\,\frac{m}{m+0.1}$
C.	concave F and G:	$F(s) = 0.02 + 0.192\,\frac{s}{s+140}$,	$G(m) = 1153\,\frac{m}{m+0.1}$
D.	concave F and G:	$F(s) = 0.2 + 1.92\,\frac{s}{s+140}$,	$G(m) = 170.3\,\frac{m}{m+0.1}$

In all systems, $s^* \approx 100$. In (A) to (C), $m^* \approx 0.1$, while in (D), $m^* \approx 1$. Figure E shows analytical plots for mode prediction and compares the protein distributions obtained by simulations of the full and reduced models.
In (A), the linearity of G makes the stochastic burst size independent of the mRNA level so that no information is lost in the course of model reduction. As a consequence, the full and reduced model yield almost identical protein distributions. Slight differences only emerge due to the temporal condensation of translation events. The modes are well predicted although F is nonlinear and its derivative is large.
In (B), the reduced model yields a slightly broader protein distribution than the full model. In Section 2.4, we have deduced the protein Fano factor of the reduced model using LNA. Obviously, it differs from the formula that is obtained for the full model if $g \neq r$. In particular, if G and, as a consequence, $\bar{G}$ is concave as in system (B) (cf. Section 4.2), so that $g < r$, the reduced model is indeed expected to have a broader distribution. Here, the effect is moderate since $\bar{G}$ is almost linear in the range of mRNA fluctuations, although G is highly nonlinear. The prediction of the mode works very well although f is large and the skewness of the distribution is great.
System (C) combines the concave transcription rate of (A) with the concave translation rate of (B). Again, the distribution of the reduced model is a bit broader than that of the full model, but the mode prediction is reliable. Nonlinearity of F does obviously not hamper the quality of model reduction.
In (D), the average mRNA level has been increased, while s^* was kept constant. This reduces the burst size, so that the mode of the protein distribution is close to its average. However, the nonlinearity of $\bar{G}$ is more pronounced than in (B) or (C). As expected, the distribution of the reduced model is significantly broader than that of the full model. Nevertheless, mode prediction works reliably.

4 Biological interpretations and applications

4.1 Extraction of burst characteristics from time course data

In the following, we will show how the burst size and frequency can be read from time course data. This helps in interpreting those measures.

4.1.1 Regulatory systems without feedback and with linear propensities

In [15], a regulatory system without feedback and with linear propensities is considered. According to that paper, the burst characteristics can be read from an idealized protein time course, where sharp increases in the protein copy number with amplitudes corresponding to α^* are followed by periods of exponential decay. The time interval between the bursts is approximately given by the inverse of the transcriptional propensity, $F(s^*)^{-1} = \frac{1}{\omega^* \cdot \nu}$.

Simulations of protein trajectories show that the extraction of the burst size is not that trivial, since the amplitude of the peaks is randomly distributed and bursts might overlap. Furthermore, a fraction of the protein molecules is instantaneously degraded during a burst, so that the actual number of produced proteins is larger than the peak height suggests. For inferring α^* from time course data, the following considerations are helpful, which are valid for all linear functions $G(m) = g \cdot m$:
Let $[\tau_0, \tau_m]$ be the lifespan of an arbitrary mRNA molecule and let $m(\tau) = 1$ for $\tau_0 \leq \tau \leq \tau_m$ (no overlap of bursts). W.l.o.g., let $\tau_0 = 0$. Moreover, let $s(0) =: s_0$. Then, for $\tau \in [\tau_0, \tau_m]$, the CME of the protein dynamics read

$$\begin{aligned} p_s(0) &= \delta_s(\{s_0\}), \\ \dot{p}_s(\tau) &= g \cdot \nu \cdot (p_{s-1} - p_s) + \nu\left((s+1)\, p_{s+1} - s\, p_s\right). \end{aligned} \tag{73}$$

The ODE of the expected value $\mathbb{E}[\, S(\tau) \,|\, 0 \leq \tau \leq \tau_m,\, S(0) = s_0 \,]$ can be formulated and explicitly solved. Evaluated at $\tau = \tau_m$, it reads:

$$\mathbb{E}[\, S(\tau) \,|\, \tau = \tau_m,\, S(0) = s_0 \,] = g - (g - s_0) \cdot e^{-\tau_m \cdot \nu}. \tag{74}$$

The lifetime of the mRNA molecule τ_m is exponentially distributed with expected value 1. At the moment of mRNA degradation, the protein copy number is thus on average given by:

$$\begin{aligned} \gamma_e^*(s_0) &:= \mathbb{E}\left[\mathbb{E}[\, S(\tau_m) \,|\, \tau_m \,] \,|\, S(0) = s_0 \,\right] = \int_0^\infty \mathbb{E}[\, S(\tau_m) \,|\, \tau_m,\, S(0) = s_0 \,] \cdot e^{-\tau_m}\, \mathrm{d}\tau_m \\ &= \frac{g \cdot \nu + s_0}{1+\nu} = \frac{\alpha^* + s_0}{1+\nu}. \end{aligned} \tag{75}$$

Eliminating the stochasticity of $S(0)$ gives

$$\gamma_e^* := \mathbb{E}[\mathbb{E}[\, \gamma_e^*(S(0)) \,|\, S(0)]] = \frac{\alpha^* + \gamma_i^*}{1+\nu} \tag{76}$$

with $\gamma_i^* := \mathbb{E}[S(0)]$. Therefore,

$$\alpha^* = \gamma_e^* \cdot (1+\nu) - \gamma_i^*. \tag{77}$$

In this context, we also define the average peak height β^*, which is the average increase of the protein level during a burst:

$$\beta^* = \frac{\alpha^*}{1+\nu}. \tag{78}$$

If ν is very small, the burst size α^* is thus almost equal to the average peak height β^*. Furthermore, when the protein level has dropped to zero before the next burst is initiated, $\beta^* = \gamma_e^* = \frac{\alpha^*}{1+\nu}$ (this often occurs[2] if $\omega^* \ll \frac{1}{\ln(\alpha^*)}$).

[2]The given boundary is derived from an idealized protein time course: When – starting from zero protein molecules – a burst of size α^* occurs at time $\tau = 0$, the mean protein amount subsequently decays according to $\alpha^* \cdot \exp(-\tau \cdot \nu)$. After the time interval $\frac{1}{\omega^* \cdot \nu}$, just before the next burst occurs, it has reached the level $\alpha^* \cdot \exp(-\frac{1}{\omega^*})$, which is smaller than 1 if $\omega^* < \frac{1}{\ln(\alpha^*)}$.

Based on Eq (75), we suggest the following
Procedure for extracting α^* and ω^* from time course data:

- From the mRNA trajectory, the length of the intervals between consecutive mRNA production events are read, and the empirical mean is calculated. This gives an estimate of $\frac{1}{\omega^* \cdot \nu}$.
- From the mRNA trajectory, overlapping bursts (where more than 1 mRNA molecule is transiently present) can be identified. Those bursts are ignored for the estimation of α^*. For all other bursts, the protein copy numbers at the moment of mRNA formation and degradation are read. These are empirical estimates for s_0 and for $\gamma_e^*(s_0)$. According to the above Eq (75), an estimate for α^* can be obtained from each non-overlapping burst. By averaging those values, the final estimate for α^* is received (Eq (77)).
- In order to obtain α^* and ω^*, ν should be known. If this is not the case, it can be inferred from degradation dynamics: When no mRNA is present, the average protein decay follows $s(\tau) = s(0) \cdot e^{-\tau \cdot \nu}$.
- As a control, the average protein level s^* can be extracted from the time course and this value can be compared to the product $\alpha^* \cdot \omega^*$.

In Table A, estimates for α^* and for ω^* obtained with this procedure are listed under the assumption that ν is known. Details on the statistical evaluation are given in Section 4.1.4. Systems (A.1) and (A.2) are linear systems without feedback. While the estimates of ω^* are accurate, α^* is always slightly underestimated. This is because overlapping bursts are eliminated, so that mRNAs with above-average lifespans that tend to produce a lot of protein are more likely to be ignored. Comparing the two systems, the average mRNA level m^* of system (A.2) is lower ($\omega^* \ll \frac{1}{\nu}$), so that overlaps are less frequent. As a consequence, the estimate for α^* is better.

4.1.2 Regulatory systems with feedback and with linear propensities

Figure F shows exemplary mRNA and protein trajectories of a system without (A) and with positive, linear feedback (B). At first glance, protein bursts in panel (B) seem to be larger and less frequent than those in panel (A). This is suprising, as the calculated local characteristics α^* and ω^* are identical in the two systems. Moreover, they correspond to the average ones: $\mathbb{E}[\alpha(M)] = \mathbb{E}\left[\frac{\nu \cdot g \cdot M}{M}\right] = \alpha^*$ and $\mathbb{E}[\omega(S)] = \mathbb{E}\left[\frac{a + f \cdot S}{\nu}\right] = \frac{a + f \cdot s^*}{\nu} = \omega^*$.
The main difference is that while the burst size $\alpha(m) = g \cdot \nu$ is independent of m in both cases, the burst frequency varies with s in the system with feedback: $\omega(s) = \frac{F(s^*)}{\nu} + \frac{F'(s^*)}{\nu}(s - s^*) = \omega^* + \frac{f}{\nu}(s - s^*)$. This means that the bursts are not homogeneous over time, but tend to accumulate and overlap if $s > s^*$, while their frequency is reduced when $s < s^*$ (the opposite holds for negative feedback). From the mRNA time course, one can tell that bursts are indeed temporally clustered so that from time to time, high mRNA copy numbers are transiently reached. Thus, the seemingly higher protein peaks do not result from larger burst sizes, but from overlapping bursts.
Our procedure to extract estimates for α^* and ω^* eliminates those overlaps, which leads to a reduced number of evaluable data and to an increased tendency to underestimate α^*. The values listed in Table A show that the estimates are still satisfying for this example, but their standard deviation increases significantly, especially for ω^* (cf. system (B)).

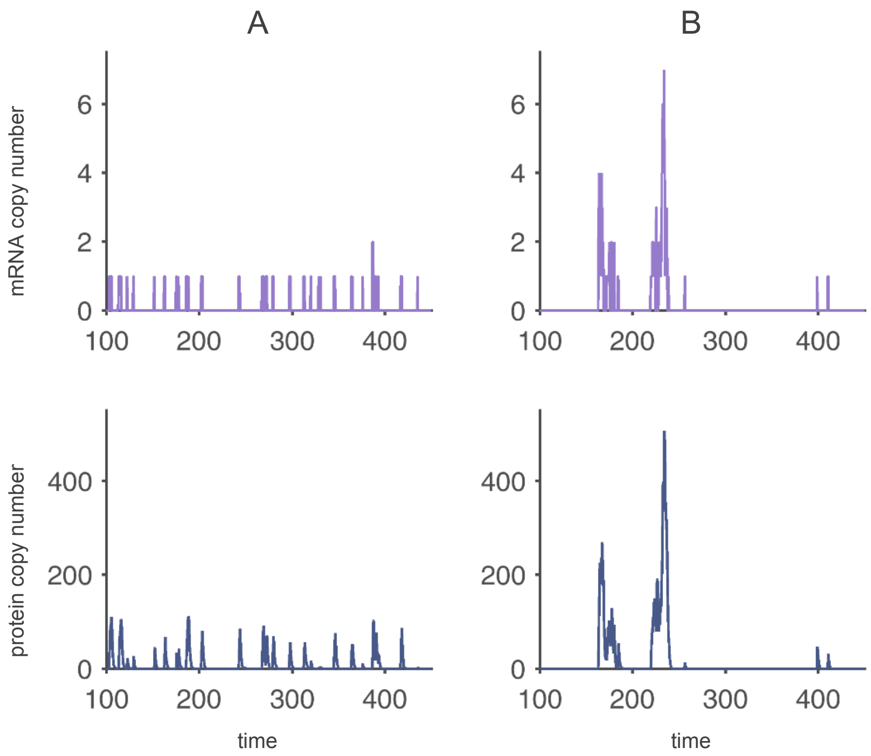

Figure F: **Simulated time courses of systems with linear propensities.** For both systems, $m^* = 0.1$, $s^* = 10$, $\alpha^* = 100$, $\omega^* = 0.1$, and $\nu = 1$. The functions F and G are given in Section 4.1.4 (cf. systems (A.1) and (B)). The upper row shows exemplary mRNA trajectories, the bottom row shows the corresponding protein trajectories. (A): System without feedback. Bursts are homogenously distributed over time. (B): System with positive, linear feedback. The temporal clustering of mRNA transcription events promotes super-bursts.

4.1.3 Regulatory systems with nonlinear propensities

If G is nonlinear, Eq (77) actually needs to be adapted to:

$$\alpha^* \cdot \frac{G(1)}{r} = G(1) \cdot \nu = \gamma_e^* \cdot (1 + \nu) - \gamma_i^*. \tag{79}$$

However, for $m^* \ll 1$, $r = \sum_{n=0}^{\infty} \frac{G(n+1)}{n+1} \frac{(m^*)^n}{n!} e^{-m^*} \approx G(1)$ (which is not the case if r is calculated with the classical deterministic model). Therefore, our evaluation method is still applicable if the average mRNA level is very low as in system (C.2) in Table A. In systems (C.1) and (D), the mRNA level is higher and therefore, the estimated burst sizes are less reliable. Estimation of ω^* is not impaired by nonlinearity of G.

In contrast, when G is linear but F is nonlinear, the estimation of α^* is reliable, while the estimate for ω^* may be inaccurate since in general, $\mathbb{E}[F(S)] \neq F(\mathbb{E}[S]) \neq F(s^*) = \omega^* \cdot \nu$. In some cases (e.g., for strictly concave or convex F), Jensen's inequality can help to qualitatively predict in which direction the estimate is biased (cf. Table A, systems (E) and (F)). In addition, it is recommended to calculate α^* from extracted values for s^* and ω^* as a control.

4.1.4 Statistical evaluation of estimates

Table A lists the estimates of various circuits. In the first set of systems, $m^* = 0.1$ and $s^* = 10$. The functions F and G are given by:

(A.1)	No feedback:	$F(s) = 0.1,$	$G(m) = 100 \cdot m$
(B)	Positive linear feedback:	$F(s) = 0.02 + 0.008 \cdot s,$	$G(m) = 100 \cdot m$
(C.1)	No feedback, convex G:	$F(s) = 0.1,$	$G(m) = 0.909 \cdot \frac{m^2}{0.01}$
(D)	No feedback, concave G:	$F(s) = 0.1,$	$G(m) = 398 \cdot \frac{m}{m+2.9}$
(E)	Negative feedback, convex F:	$F(s) = \frac{2}{s+10},$	$G(m) = 100 \cdot m$
(F)	Positive feedback, concave F:	$F(s) = 0.05 + 3 \cdot \frac{s}{s+590},$	$G(m) = 100 \cdot m$

Furthermore, two systems with $m^* = 0.01$ and $s^* = 10$ are given by:

(A.2)	No feedback:	$F(s) = 0.01,$	$G(m) = 1000 \cdot m$
(C.2)	No feedback, convex G:	$F(s) = 0.01,$	$G(m) = 9.9 \cdot \frac{m^2}{0.01}$

In order to study large and rare as well as small and frequent bursts, two values of ν have been tested (1 and 0.01). For every circuit, 10^3 mRNA and protein trajectories have been generated with the Gillespie algorithm, starting from $m = 0$ and $s = 10$ until the final reaction time $\tau = 2 \cdot 10^3$ was reached. From each time course, estimates of α^* and ω^* have been extracted, using our procedure. The values given in the table show the empirical means and SEMs of the estimates.

Table A: **Statistical evaluation of estimates of α^* and ω^* extracted from simulated mRNA and protein time course data.**

	$\nu = 1$		$\nu = 0.01$	
type	α^* $[\cdot 10^2]$	ω^* $[\cdot 10^{-1}]$	α^* $[-]$	ω^* $[\cdot 10^1]$
**analytical**	**1.00**	**1.00**	**1.00**	**1.00**
(A.1) no feedback	0.95 ± 0.04	1.00 ± 0.07	0.92 ± 0.10	1.00 ± 0.07
(B) pos. linear fb	0.84 ± 0.07	0.99 ± 0.43	0.88 ± 0.10	0.99 ± 0.40
(C.1) no fb, convex G	0.87 ± 0.04	1.00 ± 0.07	0.83 ± 0.10	1.00 ± 0.07
(D) no fb, concave G	0.97 ± 0.04	1.00 ± 0.07	0.94 ± 0.10	1.00 ± 0.07
(E) neg. fb, convex F	0.99 ± 0.04	1.40 ± 0.06	0.93 ± 0.10	1.02 ± 0.07
(F) pos. fb, concave F	0.87 ± 0.05	0.90 ± 0.13	0.90 ± 0.10	1.01 ± 0.18
**analytical**	**10.00**	**0.10**	**10.0**	**0.10**
(A.2) no feedback	9.93 ± 1.32	0.10 ± 0.02	9.86 ± 2.38	0.10 ± 0.02
(C.2) no fb, convex G	9.85 ± 1.37	0.10 ± 0.02	9.92 ± 2.42	0.11 ± 0.02

The table lists estimates of burst sizes and frequencies, extracted from simulations according to the procedure described in Section 4.1.1. The table summarizes the estimates as means ± SEMs of 10^3 repeats. Furthermore, the analytical values are given.

4.2 Noise in systems with saturated translation propensity

In order to evaluate the noise pattern in a bistable circuit that has a concave translation propensity G (or more precisely, a discrete translational propensity G which can be interpolated by a concave function) with $G(0) = 0$, we analyze the dependence of $\bar{G}'(m)$ and of $\frac{\bar{G}'(m) \cdot m}{\bar{G}(m)}$ on m. With this information, we can compare the values of g and $\frac{g}{r}$ at the two fixed points $(m_1^*, s_1^*) < (m_2^*, s_2^*)$ and thus their Fano factors $\eta(s_1^*)$ and $\eta(s_2^*)$.

First, the monotonicity of $\bar{G}'(m)$ is considered. Since G is concave,

$$G(n+1) \,\geq\, \frac{1}{2}\, G(n) \,+\, \frac{1}{2}\, G(n+2) \quad \forall\, n \in \mathbb{N}\backslash\{0\}. \tag{80}$$

holds. As a consequence,

$$\bar{G}''(m) \,=\, \sum_{n=0}^{\infty} (G(n+2) \,-\, 2G(n+1) \,+\, G(n))\, \frac{m^n}{n!}\, e^{-m} \,\leq\, 0 \quad \forall\, m \in \mathbb{R}_{\geq 0}. \tag{81}$$

Therefore, $g_1^* > g_2^*$. (In an analogous manner, one can show that $\bar{G}$ is strictly concave, convex, or strictly convex, if G is.)

Determining the monotonicity of $\frac{\bar{G}'(m) \cdot m}{\bar{G}(m)}$ is not that trivial, it depends on the choice of G. As in the main text, we set

$$G(m) = u\, \frac{m}{K_m + m}. \tag{82}$$

Then, we obtain:

$$\begin{aligned}
\frac{\bar{G}'(m) \cdot m}{\bar{G}(m)} \;&=\; \frac{\sum_{n=0}^{\infty} (G(n+1) \,-\, G(n))\, \frac{m^n}{n!}\, e^{-m}}{\sum_{n=0}^{\infty} \frac{G(n+1)}{n+1}\, \frac{m^n}{n!}\, e^{-m}} \\
&=\; \frac{\sum_{n=0}^{\infty} \frac{K_m}{(K_m+n+1)(K_m+n)}\, \frac{m^n}{n!}\, e^{-m}}{\sum_{n=0}^{\infty} \frac{1}{K_m+n+1}\, \frac{m^n}{n!}\, e^{-m}} \\
&=\; \frac{\sum_{n=0}^{\infty} h(n) \cdot h(n+1)\, \frac{m^n}{n!}\, e^{-m}}{\sum_{n=0}^{\infty} h(n+1)\, \frac{m^n}{n!}\, e^{-m}} \cdot K_m
\end{aligned} \tag{83}$$

with $h(n) := \frac{1}{K_m+n}$. Our goal is to show that the derivative of this expression with respect to m is negative. Let further $N := \sum_{n=0}^{\infty} h(n+1)\, \frac{m^n}{n!}\, e^{-m}$ be the denominator of the above expression. Then:

$$\begin{aligned}
&\frac{1}{K_m}\, \frac{d}{dm} \left(\frac{\bar{G}'(m) \cdot m}{\bar{G}(m)} \right) \\
=\;& \frac{\left(\sum_{n=0}^{\infty} (h(n+1)\, h(n+2) \,-\, h(n)\, h(n+1))\, \frac{m^n}{n!}\, e^{-m} \right) \left(\sum_{n=0}^{\infty} h(n+1)\, \frac{m^n}{n!}\, e^{-m} \right)}{N^2} \\
-\;& \frac{\left(\sum_{n=0}^{\infty} (h(n+2) \,-\, h(n+1))\, \frac{m^n}{n!}\, e^{-m} \right) \left(\sum_{n=0}^{\infty} h(n)\, h(n+1)\, \frac{m^n}{n!}\, e^{-m} \right)}{N^2}
\end{aligned}$$

$$
\begin{aligned}
= \quad & \frac{\left(\sum_{n=0}^{\infty} h(n+1)\, h(n+2)\, \frac{m^n}{n!}\, e^{-m}\right) \left(\sum_{n=0}^{\infty} h(n+1)\, \frac{m^n}{n!}\, e^{-m}\right)}{N^2} \\
- \quad & \frac{\left(\sum_{n=0}^{\infty} h(n+2)\, \frac{m^n}{n!}\, e^{-m}\right) \left(\sum_{n=0}^{\infty} h(n)\, h(n+1)\, \frac{m^n}{n!}\, e^{-m}\right)}{N^2} \\
= \quad & \frac{\left(\sum_{n=0}^{\infty} \sum_{k=0}^{n} h(k+1)\, h(k+2)\, h(n-k+1)\, \frac{m^n}{k!(n-k)!}\, e^{-2m}\right)}{N^2} \\
- \quad & \frac{\left(\sum_{n=0}^{\infty} \sum_{k=0}^{n} h(k+2)\, h(n-k)\, h(n-k+1)\, \frac{m^n}{k!(n-k)!}\, e^{-2m}\right)}{N^2} \\
= \quad & \frac{1}{N^2} \sum_{n=0}^{\infty} \sum_{k=0}^{n} h(k+2)\, h(n-k+1)\, \underbrace{[h(k+1) \,-\, h(n-k)]}_{\substack{=\frac{1}{K_m+k+1} - \frac{1}{K_m+n-k} \\ = h(k+1)\, h(n-k)\, (n-2k-1)}} \binom{n}{k}\, 2^{-n}\, \frac{(2m)^n}{n!}\, e^{-2m} \\
= \quad & \frac{1}{N^2} \sum_{n=0}^{\infty} \sum_{k=0}^{n} \underbrace{h(k+1)\, h(k+2)\, h(n-k)\, h(n-k+1)}_{:=H(k)}\, (n-k) \binom{n}{k}\, 2^{-n}\, \frac{(2m)^n}{n!}\, e^{-2m} \\
- \quad & \frac{1}{N^2} \sum_{n=0}^{\infty} \sum_{k=0}^{n} H(k)\, (k+1) \binom{n}{k}\, 2^{-n}\, \frac{(2m)^n}{n!}\, e^{-2m} \\
= \quad & \frac{1}{N^2} \sum_{n=0}^{\infty} \sum_{j=-1}^{n-1} H(j)\, (j+1) \binom{n}{j+1}\, 2^{-n}\, \frac{(2m)^n}{n!}\, e^{-2m} \\
- \quad & \frac{1}{N^2} \sum_{n=0}^{\infty} \sum_{k=0}^{n} H(k)\, (k+1) \binom{n}{k}\, 2^{-n}\, \frac{(2m)^n}{n!}\, e^{-2m} \\
= \quad & -\frac{1}{N^2} \sum_{n=0}^{\infty} H(n)\, (n+1)\, 2^{-n}\, \frac{(2m)^n}{n!}\, e^{-2m} \\
+ \quad & \frac{1}{N^2} \sum_{n=0}^{\infty} \sum_{k=0}^{n-1} H(k)\, (k+1) \left(\binom{n}{k+1} - \binom{n}{k}\right) 2^{-n}\, \frac{(2m)^n}{n!}\, e^{-2m} \\
< \quad & \frac{1}{N^2} \sum_{n=0}^{\infty} \sum_{k=0}^{\lfloor \frac{n}{2} \rfloor - 1} H(k)\, (k+1) \left(\binom{n}{k+1} - \binom{n}{k}\right) 2^{-n}\, \frac{(2m)^n}{n!}\, e^{-2m} \\
+ \quad & \frac{1}{N^2} \sum_{n=0}^{\infty} \sum_{k=\lceil \frac{n}{2} \rceil}^{n-1} H(k)\, (k+1) \left(\binom{n}{k+1} - \binom{n}{k}\right) 2^{-n}\, \frac{(2m)^n}{n!}\, e^{-2m} \\
= \quad & \frac{1}{N^2} \sum_{n=0}^{\infty} \sum_{k=0}^{\lfloor \frac{n}{2} \rfloor - 1} H(k) \cdot (k+1) \left(\binom{n}{k+1} - \binom{n}{k}\right) 2^{-n}\, \frac{(2m)^n}{n!}\, e^{-2m} \\
- \quad & \frac{1}{N^2} \sum_{n=0}^{\infty} \sum_{j=0}^{\lfloor \frac{n}{2} \rfloor - 1} H(j) \cdot (n-j) \left(\binom{n}{j+1} - \binom{n}{j}\right) 2^{-n}\, \frac{(2m)^n}{n!}\, e^{-2m}.
\end{aligned} \tag{84}
$$

During this calculation, we twice substituted $j = n - k - 1$ and used the symmetry of the binomial coefficient. Note that $H(k) = H(j)$. For $k = 0, ..., \lfloor \frac{n}{2} \rfloor - 1$, $\left(\binom{n}{k+1} - \binom{n}{k}\right) > 0$. Furthermore, $k + 1 > n - k$. Therefore, the above expression is negative.

4.3 Analyzing the robustness of stable states in bistable systems via first passage times

In Fig 5 of the main text, protein trajectories of three bistable systems (systems (A) to (C)) have been simulated. It has been shown that the robustness of the two protein expression states (ON and OFF) can be influenced by the shape of the propensity functions and by shifts in time scales. In order to give a more quantitative view on robustness, we determine first passage times (FPT) of switches from the OFF to the ON state as well as from the ON to the OFF state computationally. For each system and switching direction, 10^3 trajectories have been generated using the Gillespie algorithm, starting from either the OFF ($s^* = 20$) or the ON ($s^* = 200$) state. The first passage times τ_{FPT} are the time points when the trajectory first hits the other state. For different final reaction times τ_f, the percentage of cells for which $\tau_f > \tau_{FPT}$ holds is determined (these are the cells that have switched during $[0, \tau_f]$). The mean FPT (MFPT) of a switch is obtained by averaging the FPTs. In case a cellular state is too robust, it might be infeasible to determine the MFPT due to computational limits (infinite MFPTs are possible). Table B and Figure G summarize the results for the bistable systems from Fig 5 of the main text. One can see that in systems (A) and (B), where translation rates are linear, the inactive state is always more robust than the active state. In order to achieve the opposite, the parameters in F could be adjusted. However, the range of adjustment is limited as bistability needs to be preserved. Much greater flexibility is achieved by allowing nonlinear translation rates (system (C)) that create state-dependent burst sizes.

Table B: **Percentage of cells that have left their original state within $[0, \tau_f]$ and MFPTs**

Switch		Cells having switched during $[0, \tau_f]$ [%]				MFPT $[\cdot 10^3]$	MFPT $\cdot\, \nu$ $[\cdot 10^2]$
(A)	τ_f $[\cdot 10^3]$	2.6	5.1	10.2	20.6		
	OFF → ON	0.00	0.00	0.01	0.01	n.a.	n.a.
	ON → OFF	0.29	0.50	0.78	0.95	6.97 ± 6.06	3.49 ± 3.03
(B)	τ_f $[\cdot 10]$	1.9	3.9	7.8	15.6		
	OFF → ON	0.02	0.05	0.11	0.23	0.56 ± 0.58	2.24 ± 2.34
	ON → OFF	0.28	0.51	0.77	0.94	0.06 ± 0.05	0.22 ± 0.20
(C)	τ_f $[\cdot 10]$	3.6	7.2	14.4	28.8		
	OFF → ON	0.19	0.52	0.81	0.97	0.09 ± 0.04	0.19 ± 0.15
	ON → OFF	0.00	0.00	0.00	0.00	n.a.	n.a.

Systems (A) to (C) correspond to those from Fig 5 in the main text. OFF-to-ON switches and ON-to-OFF switches are considered separately. The percentage of cells that have undergone a transition from the initial state (OFF or ON) to the other state during $[0, \tau_f]$ is given in the middle part, depending on τ_f. The faster this percentage reaches high levels, the less robust is the state. The average and standard deviation of 10^3 independent FPTs is given in the second last column. Some states were too robust to determine the values computationally. MFPTs after normalization with respect to protein lifetime are given in the last column.

One should note that in our case, FPTs are useful for comparing the robustness of different stable states within the same regulatory system. When different systems are compared, one needs to define the time scale the comparison should be based on (e.g. the original process time, or time normalized with respect to mRNA or protein degradation rates). Due to that, we have listed the MFPTs (systems normalized with respect to mRNA lifetime) as well as the MFPTs multiplied by ν (systems normalized with respect to protein lifetime) in Table B.

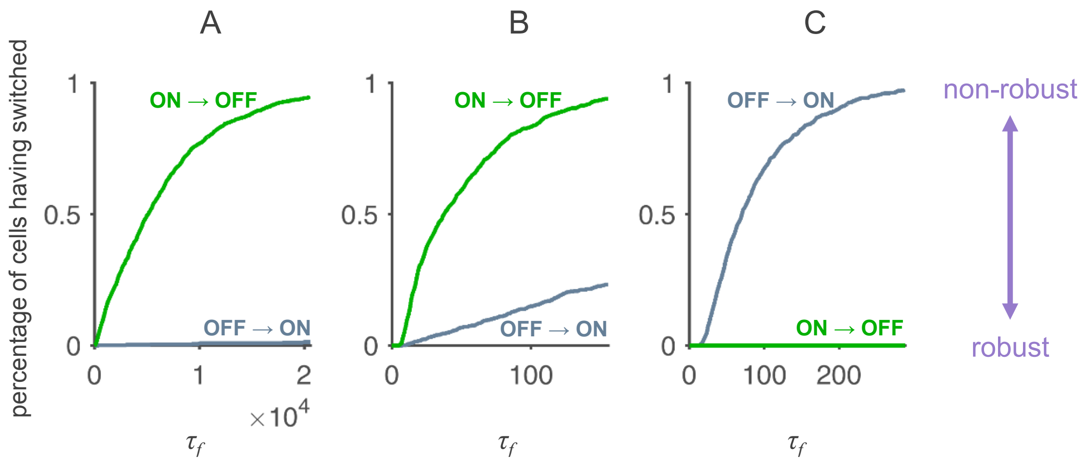

Figure G: **Visualization of Table B.** The graphs illustrate the percentage of cells that have at least once switched from their original to the other stable state within $[0, \tau_f]$ on a time scale determined by mRNA degradation. In system (C), the high expression state is more robust, in contrast to systems (A) and (B).

5 Choice of parameter values

For defining an appropriate range of parameter values, we have mainly used data on prokaryotes collected in the book "Cell Biology by the Numbers" [16].
mRNA and protein degradation rates strongly depend on the physical stability of the molecules themselves, on the physiological state of the cell, and on the presence or absence of active degradation mechanisms. Usually, the mRNA half-life is shorter than that of protein, which is why we usually chose values of $\nu < 1$. In that range, the value was varied by some orders of magnitude in order to evaluate its impact on the quality of our approaches. The average copy number of specific mRNA and protein molecules in a cell varies significantly. We have used the fact that in *E. coli*, the majority of genes have less than one mRNA molecule per cell present at any point in time, while only very few genes have 100 or more. The protein-to-mRNA ratio r was chosen to be in the order of 10 to 1000.

References

[1] van Kampen NG. Stochastic processes in physics and chemistry. 3rd ed. North-Holland; 1992.

[2] Hornos JEM, Schultz D, Innocentini GCP, Wang J, Walczak AM, Onuchic JN, et al. Self-regulating gene: An exact solution. Phys Rev E. 2005;72(5):051907. doi:10.1103/PhysRevE.72.051907.

[3] Grima R, Schmidt DR, Newman TJ. Steady-state fluctuations of a genetic feedback loop: An exact solution. J Chem Phys. 2012;137(3):035104. doi:10.1063/1.4736721.

[4] Meister A, Du C, Li YH, Wong WH. Modeling stochastic noise in gene regulatory systems. Quant Biol. 2014;2(1):1–29. doi:10.1007/s40484-014-0025-7.

[5] Tomioka R, Kimura H, J Kobayashi T, Aihara K. Multivariate analysis of noise in genetic regulatory networks. J Theor Biol. 2004;229(4):501–21. doi:10.1016/j.jtbi.2004.04.034.

[6] Walczak AM, Mugler A, Wiggins CH. Analytic methods for modeling stochastic regulatory networks. Methods Mol Biol. 2012;880:273–322. doi:10.1007/978-1-61779-833-7-13.

[7] Gillespie DT. Exact stochastic simulation of coupled chemical reactions. J Phys Chem. 1977;81(25):2340–2361. doi:10.1021/j100540a008.

[8] Zheng XD, Yang XQ, Tao Y. Bistability, probability transition rate and first-passage time in an autoactivating positive-feedback loop. PLoS One. 2011;6(3):e17104. doi:10.1371/journal.pone.0017104.

[9] Friedman N, Cai L, Xie XS. Linking stochastic dynamics to population distribution: an analytical framework of gene expression. Phys Rev Lett. 2006;97(16):168302. doi:10.1103/PhysRevLett.97.168302.

[10] Shahrezaei V, Swain PS. Analytical distributions for stochastic gene expression. Proc Natl Acad Sci U S A. 2008;105(45):17256–61. doi:10.1073/pnas.0803850105.

[11] Choi PJ, Cai L, Frieda K, Xie XS. A stochastic single-molecule event triggers phenotype switching of a bacterial cell. Science. 2008;322(5900):442–6. doi:10.1126/science.1161427.

[12] Eldar A, Elowitz MB. Functional roles for noise in genetic circuits. Nature. 2010;467(7312):167–73. doi:10.1038/nature09326.

[13] Kierzek AM, Zaim J, Zielenkiewicz P. The effect of transcription and translation initiation frequencies on the stochastic fluctuations in prokaryotic gene expression. J Biol Chem. 2001;276(11):8165–72. doi:10.1074/jbc.M006264200.

[14] Hahl SK, Kremling A. A Comparison of Deterministic and Stochastic Modeling Approaches for Biochemical Reaction Systems: On Fixed Points, Means, and Modes. Front Genet. 2016;7:157. doi:10.3389/fgene.2016.00157.

[15] Thattai M, van Oudenaarden A. Intrinsic noise in gene regulatory networks. Proc Natl Acad Sci U S A. 2001;98(15):8614–9. doi:10.1073/pnas.151588598.

[16] Milo R, Phillips R, Orme N. Cell biology by the numbers. Taylor & Francis Ltd.; 2016.
